# Supplementary material for: Progressive functional exercise versus best practice advice for adults aged 50 years or over after ankle fracture: protocol for a pilot randomised controlled trial in the UK - the Ankle Fracture Treatment: Enhancing Rehabilitation (AFTER) study
Source: BMJ Open. 2019 Nov 2;9(11):e030877. doi: 10.1136/bmjopen-2019-030877 (PMC6830709; doi:10.1136/bmjopen-2019-030877)
Supplement: Author's manuscript [file bmjopen-2019-030877.draft_revisions.pdf]

BMJ Open

BMJ Open is committed to open peer review. As part of this commitment we make the peer review history of every article we publish publicly available.

When an article is published we post the peer reviewers' comments and the authors' responses online. We also post the versions of the paper that were used during peer review. These are the versions that the peer review comments apply to.

The versions of the paper that follow are the versions that were submitted during the peer review process. They are not the versions of record or the final published versions. They should not be cited or distributed as the published version of this manuscript.

BMJ Open is an open access journal and the full, final, typeset and author-corrected version of record of the manuscript is available on our site with no access controls, subscription charges or pay-per-view fees (http://bmjopen.bmj.com).

If you have any questions on BMJ Open's open peer review process please email info.bmjopen@bmj.com

BMJ Open

Progressive functional exercise versus best practice advice for adults aged 50 years or over after ankle fracture: protocol for a pilot randomised controlled trial in the United Kingdom - the Ankle Fracture: Enhancing Rehabilitation (AFTER) study

Journal:	*BMJ Open*
Manuscript ID	bmjopen-2019-030877
Article Type:	Protocol
Date Submitted by the Author:	04-Apr-2019

Complete List of Authors:	Keene, David; University of Oxford Nuffield Department of Orthopaedics Rheumatology and Musculoskeletal Sciences,<br>Costa, Matthew; University of Oxford, Nuffield Department of Orthopaedics, Rheumatology, and Musculoskeletal Sciences<br>Tutton, Elizabeth; University of Oxford Nuffield Department of Orthopaedics Rheumatology and Musculoskeletal Sciences<br>Hopewell, Sally; University of Oxford, Nuffield Department of Orthopaedics, Rheumatology and Musculoskeletal Sciences<br>Barber, Vicki; University of Oxford Nuffield Department of Orthopaedics Rheumatology and Musculoskeletal Sciences<br>Dutton, Susan; University of Oxford, Centre for Statistics in Medicine<br>Redmond, Anthony; University of Leeds, Leeds Institute of Rheumatology and Musculoskeletal Medicine<br>Willett, Keith; University of Oxford, Orthopaedic Trauma Surgery<br>Lamb, Sarah; University of Oxford, Ndorms

Keywords:	Ankle Fractures, Clinical Trial, Geriatrics, Exercise Therapy

SCHOLARONE™ Manuscripts

Progressive functional exercise versus best practice advice for adults aged 50 years or over after ankle fracture: protocol for a pilot randomised controlled trial in the United Kingdom - the Ankle Fracture: Enhancing Rehabilitation (AFTER) study

David J. Keene[1.2*]
Matthew L. Costa[1,2]
Elizabeth Tutton[1,2,3]
Sally Hopewell[1,2,4]
Vicki S. Barber[1,2,4]
Susan J. Dutton[1,2,4]
Anthony C. Redmond[5]
Keith Willett[1,2]
Sarah E. Lamb[1,2,4]

[1] Nuffield Department of Orthopaedics, Rheumatology and Musculoskeletal Sciences, University of Oxford, Oxford, UK
[2] Oxford NIHR Biomedical Research Centre, Oxford University Hospitals NHS Foundation Trust, Oxford, UK
[3] Oxford University Hospitals NHS Foundation Trust, Oxford, UK
[4] Oxford Clinical Trials Research Unit (OCTRU), University of Oxford, Oxford, UK
[5] Leeds Institute of Rheumatic and Musculoskeletal Medicine, University of Leeds, Leeds. UK

Word count: 3941 (4000 max)

*corresponding author
Dr David J. Keene
Kadoorie Centre, Level 3, John Radcliffe Hospital, OX3 9DU
david.keene@ndorms.ox.ac.uk

For peer review only

For peer review only - http://bmjopen.bmj.com/site/about/guidelines.xhtml

ABSTRACT

Introduction

Ankle fractures result in significant morbidity in adults, with prognosis worsening with increasing age. Previous trials have not found evidence supporting supervised physiotherapy sessions, but these studies have not focused on older adults or tailored the exercise interventions to the complex needs of this patient group. The Ankle Fracture Treatment: Enhancing Rehabilitation (AFTER) study is a pilot randomised controlled trial to assess feasibility of a later definitive trial comparing best-practice advice with progressive functional exercise for adults aged 50 years and over after ankle fracture.

Methods and analysis

A multicentre pilot randomised controlled trial with an embedded qualitative study. At least forty-eight participants aged 50 years and over with an ankle fracture requiring surgical management, or non-operative management by immobilisation for at least 4 weeks, will be recruited from a minimum of three National Health Service hospitals in the United Kingdom. Participants will be allocated 1:1 via a central web-based randomisation system to: i) best-practice advice (one session of face-to-face self-management advice delivered by a physiotherapist and up to two optional additional sessions) or ii) progressive functional exercise (up to six sessions of individual face-to-face physiotherapy). An embedded qualitative study will include one-to-one interviews with up to 20 participants and a therapist focus group.

The main objectives are to assess: (i) patient engagement with the trial, measured by the participation rate; (ii) establish whether the interventions are acceptable to participants and therapists, assessed by intervention adherence levels, participant interviews and the therapist focus group; (iii) participant retention in the trial, measured by the proportion of participants providing outcome data at 6 months; (iv) acceptability of measuring outcomes at 3 and 6-month follow-up.

Ethics and dissemination

Hampshire B Research Ethics Committee (18/SC/0281) gave approval on 2nd July 2018. Results will be published in a peer-reviewed journal with authorship eligibility according to the ICMJE criteria.

For peer review only - http://bmjopen.bmj.com/site/about/guidelines.xhtml

Trial registration number

ISRCTN16612336

Keywords

Ankle Fractures, Clinical Trial, Geriatrics, Exercise Therapy

For peer review only

Article summary

Strengths and limitations of this study

- The Ankle Fracture Enhancing Rehabilitation (AFTER) study is a pilot randomised controlled trial with an embedded qualitative study recruiting at least 48 patients aged 50 years and over after an ankle fracture from at least three UK National Health Service hospitals.
- Participants will be randomly allocated to either: i) best-practice advice (one session of face-to-face self-management advice delivered by a physiotherapist and up to two more optional advice sessions) or ii) progressive functional exercise (up to six sessions of individual face-to-face physiotherapy).
- The interventions were developed using current research evidence and with input from clinical experts, researchers, and patient and public representatives.
- We aim to assess the feasibility of a future definitive randomised controlled trial in terms of patient engagement with the trial, intervention acceptability and fidelity, retention of participants in the trial, and acceptability of measuring outcomes at 3 and 6-month follow-up.
- Physiotherapists and participants are not blinded due to the nature of the interventions.

For peer review only

INTRODUCTION

Fractures may have a devastating effect on the lower limb, resulting in mobility problems and loss of independence.[1] The incidence of ankle fractures for people aged 50 years and over in the UK is 10.4 per 10,000 person-years.[2] As the proportion of ankle fractures occurring in older adults increases[3 4] and the population ages, a three-fold increase in these injuries by 2030 is projected.[5] A systematic review of outcome data after ankle fracture showed that functional outcomes worsen with increasing age,[6] likely due to lower physiological reserves (frailty), comorbidities, reduced muscle mass and power (sarcopenia),[7] and poor balance.[8] Participants in the Ankle Injury Management (AIM) trial (which recruited participants 60 years and over) self-reported an average 30% loss in ankle function, less confidence in walking, and more fear of falling from pre-injury to 6 months post-injury.[9]

In developed countries, unstable ankle fractures typically undergo internal fixation surgery to restore normal anatomy.[10] Below-knee casts are also used in older adults as they have greater risks from surgery than younger people.[11] The AIM trial showed surgery and close contact casting for managing unstable ankle fractures in older adults had equivalence in functional outcomes.[9] Whatever the initial fracture management, protective splinting (in a cast or boot) to immobilise the ankle joint and support the injured leg is commonplace. Physical impairments after ankle fracture include ankle pain, reduced ankle motion,[12] lower limb muscle strength deficits,[13 14] mobility limitations,[12 15] and walking abnormalities.[16] Many patients see a physiotherapist to aid recovery, usually in outpatient departments as most patients are ambulatory, albeit with difficulty.

A Cochrane review[17] of ankle fracture rehabilitation concluded that there was insufficient evidence to support traditional physiotherapy interventions targeting ankle joint and muscle impairments, such as stretching,[18] manual therapy,[19] and exercise.[20] Updating the Cochrane review searches in MEDLINE and EMBASE, identified another multicentre trial by Moseley and colleagues.[21] They found no differences in self-reported lower limb function or quality of life between supervised exercise and a one-off advice session for adults with ankle fractures treated surgically and conservatively. Physiotherapists delivered both interventions face-to-face. The exercises included traditional ankle exercises, single-limb weight-bearing exercises, and advice on using walking aids. The single advice session intervention had low adherence, with a third of participants obtaining one or more extra sessions of out-of-trial physiotherapy.

For peer review only - http://bmjopen.bmj.com/site/about/guidelines.xhtml

Older adults were not adequately represented in either treatment group, as the mean age was 42 years.

Physiotherapy interventions tested to date have not incorporated features of advice and exercise programmes for older adults used in other rehabilitation areas. Issues such as persistent poor balance in older adults require attention.[22] Training for functional movement problems (e.g., walking and stair climbing) and balance, rather than traditional ankle-impairment exercises, has a strong evidence base for older people's rehabilitation. Focusing on ankle impairments in rehabilitation may be insufficient for dealing with complex mobility problems in older adults after injury.[23]

The AFTER study will assess a novel approach to physiotherapy provision, progressive functional exercise. The progressive exercise programme will use contemporary evidence-based guidelines on exercise volume and load to optimise the physiological response. The widely-accepted overload principle states that movement and strength improvements require a training stimulus of sufficient volume and intensity.[24] Strong evidence suggests psychological barriers to adherence to physiotherapy advice and exercises.[25] As participants expressed fear of walking and falling in a qualitative study within the AIM trial, these psychological factors will be addressed.[26]

A pilot randomised controlled trial (RCT) is needed to investigate areas of uncertainty for a future definitive RCT, exploring trial design, recruitment, follow-up, and the acceptability of the interventions and outcome measures.

Objectives

The AFTER (Ankle Fracture Treatment: Enhancing Rehabilitation) study will assess the feasibility of a definitive RCT to compare progressive exercise with best-practice advice in patients aged 50 years or over after ankle fracture.

The main objectives of the pilot trial are to:

- Assess patient engagement with the trial, measured by the participation rate.
- Establish whether the interventions are acceptable to participants and therapists, assessed by intervention adherence levels, participant interviews and a therapist focus group.
- Determine patient retention, measured by the proportion of patients providing outcome data at 6 months

For peer review only - http://bmjopen.bmj.com/site/about/guidelines.xhtml

- Assess the acceptability of measuring outcomes at 3 and 6-month follow-up.

METHODS AND ANALYSIS

Study design

A multicentre pilot RCT with an embedded qualitative study. Participants will be allocated to either: i) best-practice advice (one session of face-to-face advice delivered by a physiotherapist, with up to two further optional advice sessions) or ii) progressive functional exercise (up to six sessions of individual face-to-face physiotherapy) (see Figure 1).

Setting

Recruitment will be from at least three NHS hospitals and their related physiotherapy services. Participants will be identified during inpatient stays or when attending fracture clinics, where they will be screened and given study information. Eligibility will be assessed by the treating clinician, usually an orthopaedic surgeon or physiotherapist.

Study participants

The target population is at least 48 adults aged 50 years or over attending NHS services to manage ankle fractures that require definitive management with surgical treatment or ankle immobilisation for at least 4 weeks.

Eligibility

We will include adults aged 50 years or over with an ankle fracture who are undergoing surgical fixation or conservative management involving ankle immobilisation for at least 4 weeks.

Patients will be excluded if they:

- are unable to adhere to trial procedures or complete questionnaires
- who do not have capacity to consent to study participation
- were not ambulatory before the injury
- are considered inappropriate for referral to physiotherapy by the clinician
- cannot attend outpatient physiotherapy at a participating centre
- have serious concomitant disease (such as terminal illness)
- have bilateral lower limb fractures

For peer review only - http://bmjopen.bmj.com/site/about/guidelines.xhtml

- have an ipsilateral concurrent Pilon fracture
- have open fracture wounds, external fixation, or substantial skin loss or grafts that would limit ankle or lower leg exercise

Recruitment

Participants will be recruited from NHS hospitals and related physiotherapy services. Posters displayed in clinics will advertise the AFTER study to patients and clinicians.

Screening and eligibility assessment

Potential participants will be inpatients or attending trauma and orthopaedic clinics. Patients presenting with an ankle fracture will be assessed against the eligibility criteria, given a copy of the participant information sheet, and asked if they wish to be considered for the study. Patients who meet the eligibility criteria and would like to participate will be approached for informed consent. If the potential participant is deemed eligible and is willing to proceed, then they and the researcher will sign and date a consent form.

Patients who do not meet the eligibility criteria or who do not wish to participate will receive standard NHS treatment. We will record the age and gender of those not eligible or who decline participation to assess the generalisability of those recruited. We will ask these patients why they declined the study and record any answers provided.

Randomisation

Consented participants will be randomised 1:1 to the intervention groups using the centralised computer randomisation service provided by the Oxford Clinical Trials Research Unit. The site's research facilitator will undertake randomisation directly themselves or will contact the study office over the telephone to access the system on their behalf. Randomisation will be computer-generated and stratified by centre and initial fracture management (surgery or non-surgical) using a variable block size to ensure participants from each centre have an equal chance of receiving each intervention. Participants will only be randomised after eligibility assessment and informed consent.

Blinding

Physiotherapists delivering the intervention and study participants will be told the treatment allocation. Researchers independent of the clinical team will collect the objective outcome measures at 6-month follow-up.

For peer review only - http://bmjopen.bmj.com/site/about/guidelines.xhtml

Interventions

Training and monitoring of intervention delivery

The best-practice advice and progressive functional exercise interventions will be provided face-to-face and one-to-one by physiotherapists. The study team will give the treating therapists training and an intervention manual on the theory and practical delivery of the interventions. Treating physiotherapists will record the delivery and content of the sessions attended by each participant.

Patient pathways vary at different hospitals so patients may have had instruction on basic ankle exercises, wound care, and use of walking aids from a physiotherapist or other health professional while an inpatient or in an acute fracture clinic before starting the study interventions. This will be recorded in the study treatment log. The intervention session(s) can be tailored to the patient's recovery level, as per usual care. Some centres make referrals for physiotherapy while patients are inpatients or at earlier clinic follow-ups. Other centres delay referrals until a clinic review several weeks later. The study will adapt screening and recruitment to embed in the patient pathway as much as is practical. The process at each centre will be reviewed as the study progresses.

The best-practice advice or progressive functional exercise sessions will be given when the participant can mobilise with unrestricted weight-bearing and do ankle exercises as guided by their surgeon or physiotherapist. We anticipate that this will usually be around 6-8 weeks post-injury.

Best-practice advice

A single advice session with a physiotherapist with one or two follow-up appointments is commonplace in the UK NHS. This was considered an acceptable provision model reflecting best-practice usual care advice in a consensus meeting with health care professionals and patient and public involvement representatives held to help design the AFTER study. There are no clinical guidelines on physiotherapy for ankle fractures. Recent NICE fracture guidelines made no specific recommendations for ankle fracture physiotherapy, but did highlight the importance of supporting advice with written information to enhance self-management.[27]

There is variation in physiotherapy provision in the NHS. However, as with many physiotherapy trials, the volume and content will be standardised to manage heterogeneity

For peer review only - http://bmjopen.bmj.com/site/about/guidelines.xhtml

and ensure a reproducible intervention that can be assessed for generalisability. This intervention should be a credible representation of current best-practice usual care advice across the NHS. Key stakeholders, including clinical experts, researchers, and patient and public representatives, contributed substantially to the development of the interventions during the design phase of the AFTER study.

The best-practice advice intervention will focus on self-management. It will include a 20- to 60-minute session (depending on local service provision) of assessment, education, reassurance, and detailed self-management advice on ankle exercises, gait training, stair climbing, walking aid advice, and basic balance exercises. An advice booklet will provide key information.

Up to two further sessions advice will be optional for participants that are having difficulties with self-management or the exercises. The physiotherapist's role will be to re-assess and re-enforce self-management advice. During the initial stage of recruitment these additional sessions may involve a telephone call or an additional face-to-face contact. In the latter stage of recruitment, these sessions will be offered as telephone consultations only. Use of additional sessions will be recorded and monitored.

Physiotherapists use a range of passive manual and electrotherapy modalities but clinical trials and systematic reviews have found limited evidence that these treatments effectively improve outcomes after ankle fracture.[17] They will therefore not be a core part of the best-practice advice but use will be recorded in the treatment logs.

Progressive functional exercise

Participants will undertake progressive functional resistance training and balance exercises. A physiotherapist will instruct on, supervise, and progress the functional exercises in up to six sessions over 16 weeks. This period allows sufficient time for neuromuscular adaptation to exercise.[28] The programme can end early if all rehabilitation goals are achieved in under six sessions. The first session will be 20-60 minutes and the rest up to 30 minutes, consistent with physiotherapy sessions in the NHS. Physiotherapists will provide assessment, advice, education about progressing recovery, gait training, walking aid instruction, and an information booklet.

The programme will be highly structured but calibrated for each individual. Tailoring programmes to the participant is a key feature of effective interventions in older adults.[29] All

For peer review only - http://bmjopen.bmj.com/site/about/guidelines.xhtml

participants will receive a core set of functional lower limb strengthening exercises in line with the evidence for improving muscular strength and power in older persons' rehabilitation.[30] Strength improvements do not necessarily translate to function and balance improvements. The programme will include supervised gait training to target major walking difficulties after ankle fracture. Gait training improves motor control during walking in older adults, which is related to functional mobility and balance improvements.[31] Balance exercises will also be included in the programme and introduced once the participant is able to weight bear sufficiently to perform these. Exercises will be practised in the clinic but conducted at home to achieve an effective dose. Based on the participant's functional goals, exercises will be progressed to make them task-specific, for example walking on uneven surfaces or slopes, climbing stairs, or jogging. The participants will receive a personal exercise guide and diary. Exercise progression will be based on evidence-based guidelines[24] but individualised by progressing and regressing the volume and load in line with each participant's capabilities and preferences.

The progressive functional exercise intervention will use simple health behaviour change techniques to optimise adherence to home exercise. We drew on the evidence-based NHS Health Trainers Handbook, recommended for routine use by health professionals to promote patient behaviour change.[32] Our group has used these techniques in other physiotherapy trials to develop feasible exercise programmes that have resulted in self-efficacy improvements.[33] The strategies use a two-stage mechanism, increasing intention to adhere to the exercise regimen and translating this behavioural intention into actual behaviour.

Participants will be asked to identify their goals following usual physiotherapy practice and, with the treating therapist's help, write an action plan for where and when they will perform their home exercises and a contingency plan for managing difficulties.

Therapists will be trained to focus on helping participants identify barriers to exercise and becoming more physically active post-injury, and facilitating problem-solving. The therapists will offer education on how exercise and physical activity can help participants to achieve their goals and will reassure participants about their capacity to exercise and increase their physical activity.[34] The intervention will give participants individualised feedback on their rehabilitation progress and reinforcement over the sessions, and will facilitate identification of barriers to doing the home exercise programme, which all have a strong evidence base to support their use in older adults.[29]

For peer review only - http://bmjopen.bmj.com/site/about/guidelines.xhtml

There is evidence that patients do not retain much of the information received face-to-face.[35] This sense of being overwhelmed by verbal instructions from physiotherapists was echoed by several patient and public involvement (PPI) group representatives, who strongly advocated quality supporting materials. A high-quality information booklet will be developed by the AFTER study team and provided to participants.

Concomitant care

Other aspects of health and social care will continue as normal. Analgesia use will be self-reported. Participants may seek other forms of treatment during follow-up, but will be asked to use their usual routes of access or referral to do so. Additional treatments, including contact with their GP or other health professionals, will be recorded in participant follow-up questionnaires.

A rigorous quality control programme will ensure intervention fidelity.[36] Participants can seek care outside the study, which will be recorded as part of health resource use. Participant crossover between intervention groups will not be allowed. The local site coordinating physiotherapist and study team will share responsibility for intervention quality control. Site visits will be conducted periodically to observe recruitment, consent, randomisation, data collection, and progressive exercise and best-practice advice sessions. Permission will be sought from the participants to observe treatment sessions. Data will be collected on intervention delivery, number of treatment sessions attended, and details about the core and adaptable components to facilitate monitoring and reporting. Sites will regularly receive feedback from quality control visits to help maintain and improve fidelity. Identified issues will be addressed by engaging the site staff in more training and increasing monitoring by the central trial team.

Outcome measures

Feasibility criteria

The main aim of this pilot RCT is to determine the feasibility of a future definitive trial.[37] The focus will not be on a primary outcome of effectiveness, but instead on meeting success criteria. The main uncertainty is whether patients find it acceptable to be randomised to different types of physiotherapy. Screening data from the AIM trial gives confidence that there are enough potential participants to investigate the feasibility criteria. To determine the feasibility of a definitive RCT, the success criteria are:

For peer review only - http://bmjopen.bmj.com/site/about/guidelines.xhtml

- A study participation rate of at least 25% of those eligible, to indicate acceptability and generalisability.
- At least 48 eligible participants across at least three sites agree to participate over a maximum of 18 months
- At least 85% of participants complete the study intervention sessions
- At least 80% of participants attend study follow-up at 6 months

Descriptions from the participant interviews and therapist focus group that indicate randomisation/interventions are acceptable will also be used to assess feasibility. Treatment logs will monitor intervention fidelity and tolerability. Adherence to home exercise will be monitored via participant self-reports. An estimate of standard deviation for the primary outcome is not required as the AIM trial will provide this information for the definitive trial.

Outcomes

Outcomes will be collected to assess the feasibility of their collection in a future definitive RCT (see Tables 1 and 2).

Patient-reported outcomes at 3 and 6 months will be:

- ankle-related symptoms and function: Olerud and Molander Ankle Score[38]
- lower-limb function limitations: Lower Extremity Functional Scale[39]
- pain: visual analogue scale (VAS), 0-100 scale
- health-related quality of life: EQ-5D-5L score[40]
- fear of falls: Falls Efficacy Scale-International (short version)[41]
- self-efficacy: self-efficacy exercise score[42]
- return to desired activities, including work, social life, and sport activities
- walking aid use and distances
- exercise adherence

At 6-month follow-up, a blinded outcome assessor will collect objective measures of ankle function and physical performance:

- ankle joint range: hand-held goniometry[43]
- muscle strength: hand-held dynamometry (Lafayette Manual Muscle Test System, Lafayette Instrument, IN, USA) of ankle dorsi/plantarflexion using a 'make' approach (working up to a maximal contraction over a maximum of 5 seconds and without

For peer review only - http://bmjopen.bmj.com/site/about/guidelines.xhtml

For peer review only

pushing into pain and the assessor maintaining position of the device).[44] Participants will be measured 3 times and have at least 10 seconds rest between attempts. Self-reported body weight will be recorded to aid interpretation of strength measures.

- mobility and balance: Short Physical Performance Battery (SPPB).[45] The test involves physical tests of balance, walking speed, and repeated rises from a chair. It has been used extensively in clinical trials owing to its practical utility, the strong evidence base for its measurement properties, and its relationship to frailty, risk of falls, and disability in older adults.[46]

Data on health resource use (consultation with primary and secondary care, prescribed and over-the-counter medication use, additional physiotherapy, and hospital admission), additional out-of-pocket expenses, and work absence (number of sickness days) will also be collected to inform a future definitive RCT. A full health economic evaluation will not be conducted. Data will be collected alongside the other outcome measures in participant questionnaires.

Adverse events

Foreseeable adverse events (AE) occurring as a result of the trial intervention(s) will be recorded. Participants will receive information on the potential AEs resulting from the treatment exercises and what they should do if they experience an AE, as would happen as part of standard NHS procedures.

Expected general side effects of any exercise, such as delayed-onset muscle soreness and temporary increases in pain <1 week, will not be recorded as AEs. Pain increases >1 week will be recorded in patient-reported questionnaires. Although unlikely, any exacerbations of other medical conditions during exercise or exercise-related injurious falls will be recorded in patient-reported questionnaires or by the site investigators if they become aware of such an event.

A serious AE (SAE) is any unexpected untoward medical occurrence related to the trial interventions that results in death, is life-threatening, requires inpatient hospitalisation or prolongation of existing hospitalisation, or results in persistent or significant disability/incapacity. SAEs are likely to be rare and are unlikely to occur as a result of the exercise programmes delivered in this study. If an SAE arises between study enrolment and final follow-up visit and is deemed related to the trial interventions, standard operating procedures will apply.

For peer review only - http://bmjopen.bmj.com/site/about/guidelines.xhtml

Follow-up data collection

Participants will be followed-up 3 months after randomisation with a postal questionnaire and 6 months after randomisation face-to-face at the hospital. They will be offered telephone, postal, or electronic follow-up if they are unable to attend the 6-month follow-up.

Participants will be asked to complete the 3-month (and, if applicable, 6-month) questionnaire(s) and return it to the AFTER trial team. Those who do not respond to the initial questionnaire will be sent at least one reminder by telephone, SMS text messaging, or email. Telephone and electronic follow-up will also be used to collect a core set of questionnaire items if these are not completed on the returned questionnaire.

Sample size

The main feasibility objective and therefore the basis of the sample size estimate is participant recruitment at three centres with a staggered start. The target sample size is a minimum of 48 participants. Based on three sites and staggered starts, this is equivalent to at least 1.5 participants per month per site over 12 months. This sample size will enable an estimate of a minimum 25% recruitment rate to within a 95% confidence interval of ±6% (calculated using the modified Wald method).[47] If the sample size is achieved sooner than 12 months, recruitment will continue up to a maximum of 60 participants to enable further feasibility assessment regarding the change to the best practice advice intervention. Recruitment will cease if the target minimum sample size is not achieved within 18 months. We have selected a conservative recruitment rate as recruitment to previous ankle fracture exercise trials in younger adults with less complex health needs has been 37-80%.[20 21]

Statistical analysis

Feasibility outcomes will be reported, including the number of participants who are approached, are eligible, consent to randomisation, and are followed-up, attendance of intervention sessions, completion rates of exercise diaries, and data completeness, all with 95% confidence intervals. Baseline characteristics will be reported using descriptive statistics, per group and overall, using mean and standard deviation (or median and interquartile range if non-normally distributed) for continuous variables and number and percentage of patients in each group for binary or categorical variables.

For peer review only - http://bmjopen.bmj.com/site/about/guidelines.xhtml

Clinical outcome measures will be reported descriptively. Differences between treatments for the intention-to-treat population will be reported with 95% confidence intervals. Withdrawals from treatment and the trial, AEs, and SAEs will be reported.

The primary statistical analysis will be carried out on the basis of intention-to-treat, with all randomised participants included and analysed according to their allocated treatment group, irrespective of which treatment they actually receive or their treatment compliance.

Embedded qualitative study

Investigating patient experience is fundamental to understanding how interventions affect patients' lives and will give insights into the acceptability of the trial interventions and randomisation to them. Patients' uncertainty when entering a trial is a recognised issue (86). It will be addressed in study training during site set-up and investigated in a nested qualitative study.

Interviews will be undertaken with a purposive sample of up to 20 participants from recruiting centres and each intervention group, approximately 4 months after randomisation. Participants will be invited to take part and provide their agreement to be contacted for an interview after they have consented to the main pilot trial. Written informed consent will be provided prior to the interview. The interviews will provide an insight into patient experience of being in the trial, the interventions, and recovery and outcomes that are important to them within the context of their life. Factors that inhibit or facilitate their ability to fully take part in the study will be used to inform a large-scale definitive RCT.

To sensitise the research team to factors that are important to study participants the semi-structured interview schedule will be informed by input from a PPI member and a therapist. Open questions will be used to ensure participants can talk freely about what is important to them. Participants will choose whether to be interviewed in the local hospital, over the telephone, or at home.

The experiences of therapists delivering the trial interventions and recruiting patients will also be explored. Therapists will be invited to participate in a focus group to be held at the end of the recruitment phase.

Interviews and the focus group will be audio-recorded, transcribed verbatim and NVivo (QSR International Pty Ltd, Melbourne, Australia) will be used to help manage the data. Data

For peer review only - http://bmjopen.bmj.com/site/about/guidelines.xhtml

will be coded and grouped into categories and themes drawing on thematic analysis.[48] Any quotes used in reporting of the findings will be anonymised.

PATIENT AND PUBLIC INVOLVEMENT

The development of the study funding application, intervention development and study materials were supported by a patient and public involvement group, who will also be involved in developing the dissemination strategy.

ETHICS AND DISSEMINATION

The study has been approved by the South Central – Hampshire B Research Ethics Committee (Ref: 18/SC/0281) and the Health Research Authority. It has been prospectively registered (ISRCTN16612336). All protocol amendments are will be subject to review by the Sponsor (Oxford University) the ethics committee and Health Research Authority and will be included in the final report. All data will be processed following Oxford Clinical Trials Research Unit (OCTRU) standard operating procedures. Adverse events and serious adverse events are likely to be rare. If they occur they will be reviewed by the trial management group.

This protocol has been reported following the Standard Protocol Items: Recommendations for Interventional Trials (SPIRIT) statement.[49] Results will be published in an open-access journal, reported following the Consolidated Standards of Reporting Trials (CONSORT) guidelines for pilot and feasibility trials.[50 51] The Template for Intervention Description and Replication (TIDieR) statement will be used to report the intervention,[52] ensuring replication is possible. Participants will be asked if and how they would like to be informed of the study results during the consent process. We will share study results before publication with those participants who request it.

For peer review only

For peer review only - http://bmjopen.bmj.com/site/about/guidelines.xhtml

Acknowledgements

The AFTER study collaborating Principal Investigators are:

Hannah Perkins (Oxford University Hospitals NHS Foundation Trust)

Carol McCrum (East Sussex Healthcare NHS Trust)

Jacky Jones (Guy's and St Thomas' NHS Foundation Trust)

Carey McClellan (University Hospitals NHS Foundation Trust)

Fiona Cowell (Royal Liverpool and Broadgreen University Hospital NHS Trust)

We acknowledge English language editing by Dr Jennifer A de Beyer of the Centre for Statistics in Medicine (University of Oxford).

Author contributions

DJK is the chief investigator and drafted the manuscript. All authors were involved in the study design and have reviewed and approved the manuscript.

Funding statement

This report is independent research supported by the National Institute for Health Research (NIHR Post-Doctoral Fellowship, Dr David Keene, PDF-2016-09-056). The report was supported by the NIHR Biomedical Research Centre, Oxford. Professor Lamb receives funding from the NIHR Collaboration for Leadership in Applied Health Research and Care Oxford at Oxford Health NHS Foundation Trust.

Disclaimer

The views expressed in this publication are those of the author(s) and not necessarily those of the NHS, the National Institute for Health Research, or the Department of Health.

The study sponsor and funders had no role in study design; writing of the report; and the decision to submit the report for publication.

Data statement

Data sharing request procedures will be reported in the published results manuscript.

Competing interests statement

For peer review only

None declared.

For peer review only

References

1. Pasco JA, Sanders KM, Hoekstra FM, et al. The human cost of fracture. *Osteoporos Int* 2005;16(12):2046-52.
2. Curtis EM, van der Velde R, Moon RJ, et al. Epidemiology of Fractures in the United Kingdom 1988-2012: Variation with age, sex, geography, ethnicity and socioeconomic status. *Bone* 2016;87:19-26. doi: 10.1016/j.bone.2016.03.006
3. Thur CK, Edgren G, Jansson KA, et al. Epidemiology of adult ankle fractures in Sweden between 1987 and 2004: A population-based study of 91,410 Swedish inpatients. *Acta Orthop* 2012;83(3):276-81. doi: 10.3109/17453674.2012.672091
4. Court-Brown CM, Caesar B. Epidemiology of adult fractures: A review. *Injury* 2006;37(8):691-7. doi: 10.1016/j.injury.2006.04.130
5. Kannus P, Palvanen M, Niemi S, et al. Stabilizing incidence of low-trauma ankle fractures in elderly people Finnish statistics in 1970-2006 and prediction for the future. *Bone* 2008;43(2):340-42. doi: S8756-3282(08)00225-1 [pii]

10.1016/j.bone.2008.04.015
6. Beckenkamp PR, Lin CW, Chagpar S, et al. Prognosis of physical function following ankle fracture: a systematic review with meta-analysis. *J Orthop Sports Phys Ther* 2014;44(11):841-51, B2. doi: 10.2519/jospt.2014.5199
7. Clegg A, Young J, Iliffe S, et al. Frailty in elderly people. *Lancet* 2013;381(9868):752-62. doi: 10.1016/S0140-6736(12)62167-9
8. Seeley DG, Kelsey J, Jergas M, et al. Predictors of ankle and foot fractures in older women. The Study of Osteoporotic Fractures Research Group. *J Bone Miner Res* 1996;11(9):1347-55. doi: 10.1002/jbmr.5650110920
9. Keene DJ, Mistry D, Nam J, et al. The Ankle Injury Management (AIM) Trial: A pragmatic, multi-centre, equivalence randomised controlled trial and economic evaluation comparing close contact casting with open surgical reduction and internal fixation in the treatment of unstable ankle fractures in patients over 60 years. *Health Technol Assess*;In press
10. Handley R, Gandhe A. Ankle fractures. In: Bulstrode CJK, ed. Oxford textbook of trauma and orthopaedics. 2nd ed. Oxford: Oxford University Press 2011.
11. Willett K, Keene DJ, Morgan L, et al. Ankle Injury Management (AIM): design of a pragmatic multi-centre equivalence randomised controlled trial comparing Close Contact Casting (CCC) to Open surgical Reduction and Internal Fixation (ORIF) in the treatment of unstable ankle fractures in patients over 60 years. *BMC Musculoskelet Disord* 2014;15(1):79. doi: 10.1186/1471-2474-15-79
12. Lin CW, Moseley AM, Herbert RD, et al. Pain and dorsiflexion range of motion predict short- and medium-term activity limitation in people receiving physiotherapy intervention after ankle fracture: an observational study. *Aust J Physiother* 2009;55(1):31-7.
13. Stevens JE, Walter GA, Okereke E, et al. Muscle adaptations with immobilization and rehabilitation after ankle fracture. *Med Sci Sports Exerc* 2004;36(10):1695-701.
14. Psatha M, Wu Z, Gammie FM, et al. A longitudinal MRI study of muscle atrophy during lower leg immobilization following ankle fracture. *J Magn Reson Imaging* 2012;35(3):686-95. doi: 10.1002/jmri.22864
15. Hancock MJ, Herbert RD, Stewart M. Prediction of outcome after ankle fracture. *J Orthop Sports Phys Ther* 2005;35(12):786-92.
16. Wolf SI, Lessl E, Müller S, et al. Persistent diminished joint function following ankle fracture - a risk factor for posttraumatic osteoarthritis? [abstract]. *Gait Posture* 2013;38 (Suppl 1):S101-02. doi: http://dx.doi.org/10.1016/j.gaitpost.2013.07.207

For peer review only - http://bmjopen.bmj.com/site/about/guidelines.xhtml

17. Lin CW, Donkers NA, Refshauge KM, et al. Rehabilitation for ankle fractures in adults. *Cochrane Database Syst Rev* 2012;11:Cd005595. doi: 10.1002/14651858.CD005595.pub3
18. Moseley AM, Herbert RD, Nightingale EJ, et al. Passive stretching does not enhance outcomes in patients with plantarflexion contracture after cast immobilization for ankle fracture: a randomized controlled trial. *Arch Phys Med Rehabil* 2005;86(6):1118-26. doi: 10.1016/j.apmr.2004.11.017
19. Lin CW, Moseley AM, Haas M, et al. Manual therapy in addition to physiotherapy does not improve clinical or economic outcomes after ankle fracture. *J Rehabil Med* 2008;40(6):433-9. doi: 10.2340/16501977-0187
20. Nilsson GM, Jonsson K, Ekdahl CS, et al. Effects of a training program after surgically treated ankle fracture: a prospective randomised controlled trial. *BMC Musculoskelet Disord* 2009;10:118. doi: 10.1186/1471-2474-10-118
21. Moseley AM, Beckenkamp PR, Haas M, et al. Rehabilitation After Immobilization for Ankle Fracture: The EXACT Randomized Clinical Trial. *JAMA* 2015;314(13):1376-85. doi: 10.1001/jama.2015.12180
22. Dudek K, Druzbicki M, Przysada G, et al. Assessment of standing balance in patients after ankle fractures. *Acta Bioeng Biomech* 2014;16(4):59-65.
23. Ebrahim S, Adamson J, Ayis S, et al. Locomotor disability: Meaning, causes and effects of interventions. *J Health Serv Res Policy* 2008;13 Suppl 3:38-46. doi: 13/suppl_3/38 [pii]

10.1258/jhsrp.2008.008013
24. Kraemer W, Adams K, Cafarelli E, et al. American College of Sports Medicine position stand. Progression models in resistance training for healthy adults. *Med Sci Sports Exerc* 2002;34(2):364-80.
25. Jack K, McLean SM, Moffett JK, et al. Barriers to treatment adherence in physiotherapy outpatient clinics: a systematic review. *Man Ther* 2010;15(3):220-8. doi: 10.1016/j.math.2009.12.004
26. Ayis S, Gooberman-Hill R, Bowling A, et al. Predicting catastrophic decline in mobility among older people. *Age Ageing* 2006;35(4):382-87. doi: 10.1093/ageing/afl004
27. National Institute for Clinical and Care Excellence. Draft for consultation: Fractures (non-complex): assessment and management: National Clinical Guideline Centre 2015.
28. Byrne C, Faure C, Keene DJ, et al. Ageing, muscle power, and physical function: A systematic review and implications for pragmatic training interventions. *Sports Med* 2016;In Press
29. Zubala A, MacGillivray S, Frost H, et al. Promotion of physical activity interventions for community dwelling older adults: A systematic review of reviews. *PLoS One* 2017;12(7):e0180902. doi: 10.1371/journal.pone.0180902
30. National Institute for Clinical and Care Excellence. Falls in older people: Assessing risk and prevention. Manchester: NICE 2013.
31. Brach JS, Lowry K, Perera S, et al. Improving motor control in walking: A randomized clinical trial in older adults with subclinical walking difficulty. *Arch Phys Med Rehabil* 2015;96(3):388-94. doi: 10.1016/j.apmr.2014.10.018
32. Michie S, Rumsey N, Fussell A, et al. Improving health: Changing behaviour.: NHS health trainer handbook, 2008.
33. Lamb SE, Williamson EM, Heine PJ, et al. Exercises to improve function of the rheumatoid hand (SARAH): A randomised controlled trial. *Lancet* 2015;385(9966):421-29. doi: 10.1016/S0140-6736(14)60998-3
34. Dobson F, Bennell KL, French SD, et al. Barriers and Facilitators to Exercise Participation in People with Hip and/or Knee Osteoarthritis: Synthesis of the Literature Using

For peer review only - http://bmjopen.bmj.com/site/about/guidelines.xhtml

Behavior Change Theory. *Am J Phys Med Rehabil* 2016;95(5):372-89. doi: 10.1097/phm.0000000000000448
35. Kessels RPC. Patients' memory for medical information. *J R Soc Med* 2003;96(5):219-22.
36. Hasson H. Invervention fidelity in clinical trials. In: Richards DA, Hallberg IR, eds. Complex interventions in health. Abingdon: Routledge 2015.
37. Thabane L, Ma J, Chu R, et al. A tutorial on pilot studies: The what, why and how. *BMC Med Res Methodol* 2010;10:1. doi: 10.1186/1471-2288-10-1
38. Olerud C, Molander H. A scoring scale for symptom evaluation after ankle fracture. *Arch Orthop Trauma Surg* 1984;103(3):190-4.
39. Binkley JM, Stratford PW, Lott SA, et al. The Lower Extremity Functional Scale (LEFS): Scale Development, Measurement Properties, and Clinical Application. *Phys Ther* 1999;79(4):371-83. doi: 10.1093/ptj/79.4.371
40. Brooks R. EuroQol: the current state of play. *Health Policy* 1996;37(1):53-72.
41. Kempen GIJM, Yardley L, Van Haastregt JCM, et al. The Short FES-I: a shortened version of the falls efficacy scale-international to assess fear of falling. *Age and Ageing* 2008;37(1):45-50. doi: 10.1093/ageing/afm157
42. Resnick B, Jenkins LS. Testing the Reliability and Validity of the Self-Efficacy for Exercise Scale. *Nurs Res* 2000;49(3):154-59.
43. Bennell KL, Talbot RC, Wajswelner H, et al. Intra-rater and inter-rater reliability of a weight-bearing lunge measure of ankle dorsiflexion. *Aust J Physiother* 1998;44(3):175-80.
44. Wang C-Y, Olson SL, Protas EJ. Test-retest strength reliability: hand-held dynamometry in community-dwelling elderly fallers. *Arch Phys Med Rehabil* 2002;83(6):811-15.
45. Guralnik JM, Simonsick EM, Ferrucci L, et al. A short physical performance battery assessing lower extremity function: Association with self-reported disability and prediction of mortality and nursing home admission. *J Gerontol* 1994;49(2):M85-M94. doi: 10.1093/geronj/49.2.M85
46. Freiberger E, de Vreede P, Schoene D, et al. Performance-based physical function in older community-dwelling persons: A systematic review of instruments. *Age Ageing* 2012;41(6):712-21. doi: 10.1093/ageing/afs099
47. Agresti A, Coull BA. Approximate is better than "exact" for interval estimation of binomial proportions. *Am Stat* 1998;52(2):119-26. doi: 10.1080/00031305.1998.10480550
48. Braun V, Clarke V. Using thematic analysis in psychology. *Qual Res Psychol* 2006;3(2):77-101. doi: 10.1191/1478088706qp063oa
49. Chan AW, Tetzlaff JM, Gotzsche PC, et al. SPIRIT 2013 explanation and elaboration: guidance for protocols of clinical trials. *Bmj* 2013;346:e7586. doi: 10.1136/bmj.e7586
50. Schulz KF, Altman DG, Moher D. CONSORT 2010 statement: updated guidelines for reporting parallel group randomised trials. *Bmj* 2010;340:c332. doi: 10.1136/bmj.c332
51. Eldridge SM, Chan CL, Campbell MJ, et al. CONSORT 2010 statement: extension to randomised pilot and feasibility trials. *BMJ* 2016;355:i5239. doi: 10.1136/bmj.i5239
52. Hoffmann TC, Glasziou PP, Boutron I, et al. Better reporting of interventions: template for intervention description and replication (TIDieR) checklist and guide. *Bmj* 2014;348:g1687. doi: 10.1136/bmj.g1687

For peer review only - http://bmjopen.bmj.com/site/about/guidelines.xhtml

Figure 1: Study flow diagram for the AFTER study

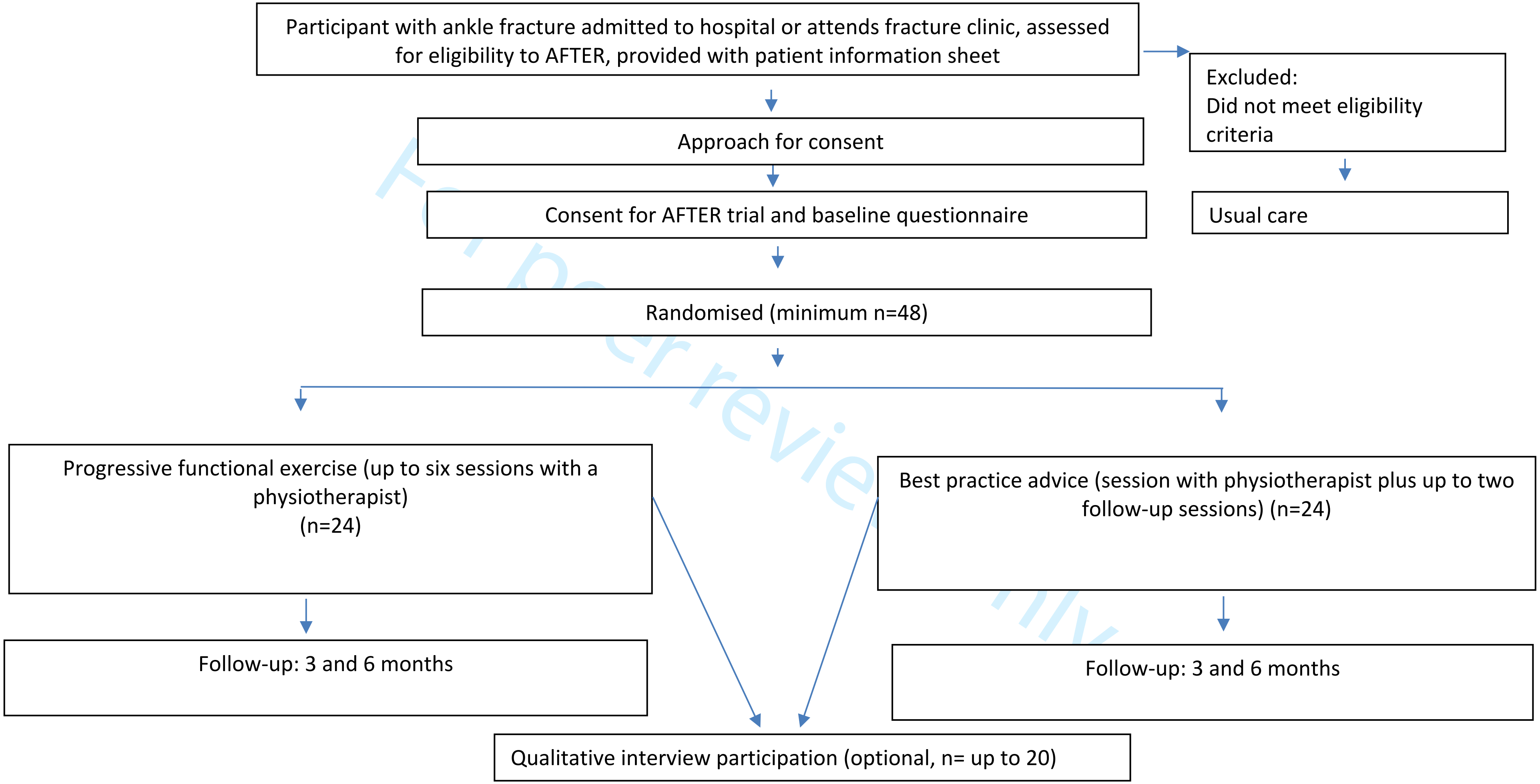

Table 1: Time points at which the outcomes will be assessed

TIMEPOINT	Enrolment	Allocation	0-4 months	3-month follow up	6-month follow up
Screening log	✓				
Informed consent	✓				
Eligibility confirmed	✓				
Randomisation		✓			
Control: best practice advice			1 physio session (up to 2 more if struggling)		
Intervention: Progressive functional exercise			Up to 6 physio sessions		
Baseline questionnaire	✓				
Follow-up questionnaire				✓	✓
Follow-up clinic visit at hospital					✓
Follow-up reminders				✓	✓
Qualitative interview (optional)			✓		

For peer review only - http://bmjopen.bmj.com/site/about/guidelines.xhtml

Table 2: Participant timeline

Outcome	Measurement	Time point
Demographic	age, gender, height, weight, ethnicity, smoking, alcohol consumption, date of injury, fracture classification, initial fracture management, pre-injury walking aid use and exercise tolerance, current work status, level of education, place of residence, social support	Baseline
Ankle-related symptoms and function	Olerud and Molander Ankle Score[38]	Baseline, 3 and 6 months
Lower limb function limitations	Lower Extremity Functional Scale	Baseline, 3 and 6 months
Pain	Visual Analogue Scale (VAS)	Baseline, 3 and 6 months
Health-related quality of life	EQ-5D-5L score[40]	Baseline, 3 and 6 months
Fear of falls	Falls Efficacy Scale-International (short)	Baseline, 3 and 6 months
Self-efficacy	Self-efficacy exercise score[42]	3 and 6 months
Return to desired activities, including work, social life and sport activities	Patient-reported return to activities	3 and 6 months
Walking aid use and distance	Patient-reported use of aids and maximum distance walked on any single occasion	Baseline (recall pre-injury and current status), 3 and 6 months
Adherence to exercise	Patient-reported exercise performance	3 and 6 months
Mobility and balance	Short Physical Performance Battery (SPPB)[45]	6 months
Ankle joint range	Hand-held goniometry[43]	6 months
Muscle strength	Hand-held dynamometry	6 months
Medication usage	Prescribed and over-the-counter medications	Baseline, 3 and 6 months
Work disability	Sick leave (days)	Baseline, 3 and 6 months
Healthcare use	NHS outpatient and community services (e.g., GP, additional physical therapy)<br>NHS inpatient and day case (e.g., radiography, readmissions)	3 and 6 months

For peer review only - http://bmjopen.bmj.com/site/about/guidelines.xhtml

Outcome	Measurement	Time point
	Private health care services	
Out-of-pocket expenses	Patient-related out-of-pocket expenses recording form	3 and 6 months
Adverse events	Patient-reported adverse events	3 and 6 months

For peer review only

SPIRIT

STANDARD PROTOCOL ITEMS: RECOMMENDATIONS FOR INTERVENTIONAL TRIALS

SPIRIT 2013 Checklist: Recommended items to address in a clinical trial protocol and related documents*

Section/item	ItemNo	Description	Reported
**Administrative information**			
Title	1	Descriptive title identifying the study design, population, interventions, and, if applicable, trial acronym	Y
Trial registration	2a	Trial identifier and registry name. If not yet registered, name of intended registry	Y
	2b	All items from the World Health Organization Trial Registration Data Set	Y
Protocol version	3	Date and version identifier	Publication is current version
Funding	4	Sources and types of financial, material, and other support	Y
Roles and responsibilities	5a	Names, affiliations, and roles of protocol contributors	Y
	5b	Name and contact information for the trial sponsor	Y – Oxford University

For peer review only

	5c	Role of study sponsor and funders, if any, in study design; collection, management, analysis, and interpretation of data; writing of the report; and the decision to submit the report for publication, including whether they will have ultimate authority over any of these activities	Y – disclaimer section
	5d	Composition, roles, and responsibilities of the coordinating centre, steering committee, endpoint adjudication committee, data management team, and other individuals or groups overseeing the trial, if applicable (see Item 21a for data monitoring committee)	Y – adverse events section
**Introduction**			
Background and rationale	6a	Description of research question and justification for undertaking the trial, including summary of relevant studies (published and unpublished) examining benefits and harms for each intervention	Y
	6b	Explanation for choice of comparators	Y
Objectives	7	Specific objectives or hypotheses	Y

For peer review only

Trial design	8	Description of trial design including type of trial (eg, parallel group, crossover, factorial, single group), allocation ratio, and framework (eg, superiority, equivalence, noninferiority, exploratory)	Y

Methods: Participants, interventions, and outcomes

Study setting	9	Description of study settings (eg, community clinic, academic hospital) and list of countries where data will be collected. Reference to where list of study sites can be obtained	Y
Eligibility criteria	10	Inclusion and exclusion criteria for participants. If applicable, eligibility criteria for study centres and individuals who will perform the interventions (eg, surgeons, psychotherapists)	Y
Interventions	11a	Interventions for each group with sufficient detail to allow replication, including how and when they will be administered	Y

For peer review only - http://bmjopen.bmj.com/site/about/guidelines.xhtml

	11b	Criteria for discontinuing or modifying allocated interventions for a given trial participant (eg, drug dose change in response to harms, participant request, or improving/worsening disease)	Y
	11c	Strategies to improve adherence to intervention protocols, and any procedures for monitoring adherence (eg, drug tablet return, laboratory tests)	Y
	11d	Relevant concomitant care and interventions that are permitted or prohibited during the trial	Y
Outcomes	12	Primary, secondary, and other outcomes, including the specific measurement variable (eg, systolic blood pressure), analysis metric (eg, change from baseline, final value, time to event), method of aggregation (eg, median, proportion), and time point for each outcome. Explanation of the clinical relevance of chosen efficacy and harm outcomes is strongly recommended	Y

Participant timeline	13	Time schedule of enrolment, interventions (including any run-ins and washouts), assessments, and visits for participants. A schematic diagram is highly recommended (see Figure)	Y
Sample size	14	Estimated number of participants needed to achieve study objectives and how it was determined, including clinical and statistical assumptions supporting any sample size calculations	Y
Recruitment	15	Strategies for achieving adequate participant enrolment to reach target sample size	Y

Methods: Assignment of interventions (for controlled trials)

Allocation:

Sequence generation	16a	Method of generating the allocation sequence (eg, computer-generated random numbers), and list of any factors for stratification. To reduce predictability of a random sequence, details of any planned restriction (eg, blocking) should be provided in a separate document that is unavailable to those who enrol participants or assign interventions	Y
Allocation concealment mechanism	16b	Mechanism of implementing the allocation sequence (eg, central telephone; sequentially numbered, opaque, sealed envelopes), describing any steps to conceal the sequence until interventions are assigned	Y
Implementation	16c	Who will generate the allocation sequence, who will enrol participants, and who will assign participants to interventions	Y

Blinding (masking)	17a	Who will be blinded after assignment to interventions (eg, trial participants, care providers, outcome assessors, data analysts), and how	Y
	17b	If blinded, circumstances under which unblinding is permissible, and procedure for revealing a participant's allocated intervention during the trial	N/A

Methods: Data collection, management, and analysis

Data collection methods	18a	Plans for assessment and collection of outcome, baseline, and other trial data, including any related processes to promote data quality (eg, duplicate measurements, training of assessors) and a description of study instruments (eg, questionnaires, laboratory tests) along with their reliability and validity, if known. Reference to where data collection forms can be found, if not in the protocol	Y

	18b	Plans to promote participant retention and complete follow-up, including list of any outcome data to be collected for participants who discontinue or deviate from intervention protocols	Y
Data management	19	Plans for data entry, coding, security, and storage, including any related processes to promote data quality (eg, double data entry; range checks for data values). Reference to where details of data management procedures can be found, if not in the protocol	Y
Statistical methods	20a	Statistical methods for analysing primary and secondary outcomes. Reference to where other details of the statistical analysis plan can be found, if not in the protocol	Y
	20b	Methods for any additional analyses (eg, subgroup and adjusted analyses)	N/A
	20c	Definition of analysis population relating to protocol non-adherence (eg, as randomised analysis), and any statistical methods to handle missing data (eg, multiple imputation)	N/A

For peer review only - http://bmjopen.bmj.com/site/about/guidelines.xhtml

Methods: Monitoring

Data monitoring	21a	Composition of data monitoring committee (DMC); summary of its role and reporting structure; statement of whether it is independent from the sponsor and competing interests; and reference to where further details about its charter can be found, if not in the protocol. Alternatively, an explanation of why a DMC is not needed	N/A – feasibility study
	21b	Description of any interim analyses and stopping guidelines, including who will have access to these interim results and make the final decision to terminate the trial	N/A
Harms	22	Plans for collecting, assessing, reporting, and managing solicited and spontaneously reported adverse events and other unintended effects of trial interventions or trial conduct	Y
Auditing	23	Frequency and procedures for auditing trial conduct, if any, and whether the process will be independent from investigators and the sponsor	Y

For peer review only - http://bmjopen.bmj.com/site/about/guidelines.xhtml

Ethics and dissemination

Research ethics approval	24	Plans for seeking research ethics committee/institutional review board (REC/IRB) approval	Y
Protocol amendments	25	Plans for communicating important protocol modifications (eg, changes to eligibility criteria, outcomes, analyses) to relevant parties (eg, investigators, REC/IRBs, trial participants, trial registries, journals, regulators)	Y
Consent or assent	26a	Who will obtain informed consent or assent from potential trial participants or authorised surrogates, and how (see Item 32)	Y
	26b	Additional consent provisions for collection and use of participant data and biological specimens in ancillary studies, if applicable	N/A
Confidentiality	27	How personal information about potential and enrolled participants will be collected, shared, and maintained in order to protect confidentiality before, during, and after the trial	Y

For peer review only

Declaration of interests	28	Financial and other competing interests for principal investigators for the overall trial and each study site	Y
Access to data	29	Statement of who will have access to the final trial dataset, and disclosure of contractual agreements that limit such access for investigators	Y
Ancillary and post-trial care	30	Provisions, if any, for ancillary and post-trial care, and for compensation to those who suffer harm from trial participation	N/A
Dissemination policy	31a	Plans for investigators and sponsor to communicate trial results to participants, healthcare professionals, the public, and other relevant groups (eg, via publication, reporting in results databases, or other data sharing arrangements), including any publication restrictions	Y
	31b	Authorship eligibility guidelines and any intended use of professional writers	Y

For peer review only

	31c	Plans, if any, for granting public access to the full protocol, participant-level dataset, and statistical code	Y – data statement, this publication will make the protocol opn access
**Appendices**			
Informed consent materials	32	Model consent form and other related documentation given to participants and authorised surrogates	Not included in the protocol manuscript
Biological specimens	33	Plans for collection, laboratory evaluation, and storage of biological specimens for genetic or molecular analysis in the current trial and for future use in ancillary studies, if applicable	N/A

*It is strongly recommended that this checklist be read in conjunction with the SPIRIT 2013 Explanation & Elaboration for important clarification on the items. Amendments to the protocol should be tracked and dated. The SPIRIT checklist is copyrighted by the SPIRIT Group under the Creative Commons "Attribution-NonCommercial-NoDerivs 3.0 Unported" license.

For peer review only

BMJ Open

Progressive functional exercise versus best practice advice for adults aged 50 years or over after ankle fracture: protocol for a pilot randomised controlled trial in the United Kingdom - the Ankle Fracture: Enhancing Rehabilitation (AFTER) study

Journal:	*BMJ Open*
Manuscript ID	bmjopen-2019-030877.R1
Article Type:	Protocol
Date Submitted by the Author:	03-Sep-2019

Complete List of Authors:	Keene, David; University of Oxford Nuffield Department of Orthopaedics Rheumatology and Musculoskeletal Sciences,<br>Costa, Matthew; University of Oxford, Nuffield Department of Orthopaedics, Rheumatology, and Musculoskeletal Sciences<br>Tutton, Elizabeth; University of Oxford Nuffield Department of Orthopaedics Rheumatology and Musculoskeletal Sciences<br>Hopewell, Sally; University of Oxford, Nuffield Department of Orthopaedics, Rheumatology and Musculoskeletal Sciences<br>Barber, Vicki; University of Oxford Nuffield Department of Orthopaedics Rheumatology and Musculoskeletal Sciences<br>Dutton, Susan; University of Oxford, Centre for Statistics in Medicine<br>Redmond, Anthony; University of Leeds, Leeds Institute of Rheumatology and Musculoskeletal Medicine<br>Willett, Keith; University of Oxford, Orthopaedic Trauma Surgery<br>Lamb, Sarah; University of Oxford, Ndorms

**Primary Subject Heading**:	Rehabilitation medicine
Secondary Subject Heading:	Geriatric medicine, Surgery
Keywords:	Ankle Fractures, Clinical Trial, Geriatrics, Exercise Therapy

SCHOLARONE™ Manuscripts

Progressive functional exercise versus best practice advice for adults aged 50 years or over after ankle fracture: protocol for a pilot randomised controlled trial in the United Kingdom - the Ankle Fracture: Enhancing Rehabilitation (AFTER) study

David J. Keene[1.2*]
Matthew L. Costa[1,2]
Elizabeth Tutton[1,2,3]
Sally Hopewell[1,2,4]
Vicki S. Barber[1,2,4]
Susan J. Dutton[1,2,4]
Anthony C. Redmond[5]
Keith Willett[1,2]
Sarah E. Lamb[1,2,4]

[1] Nuffield Department of Orthopaedics, Rheumatology and Musculoskeletal Sciences, University of Oxford, Oxford, UK
[2] Oxford NIHR Biomedical Research Centre, Oxford University Hospitals NHS Foundation Trust, Oxford, UK
[3] Oxford University Hospitals NHS Foundation Trust, Oxford, UK
[4] Oxford Clinical Trials Research Unit (OCTRU), University of Oxford, Oxford, UK
[5] Leeds Institute of Rheumatic and Musculoskeletal Medicine, University of Leeds, Leeds. UK

Word count: 3941 (4000 max)

*corresponding author
Dr David J. Keene
Kadoorie Centre, Level 3, John Radcliffe Hospital, OX3 9DU
david.keene@ndorms.ox.ac.uk

For peer review only

ABSTRACT

Introduction

Ankle fractures result in significant morbidity in adults, with prognosis worsening with increasing age. Previous trials have not found evidence supporting supervised physiotherapy sessions, but these studies have not focused on older adults or tailored the exercise interventions to the complex needs of this patient group. The Ankle Fracture Treatment: Enhancing Rehabilitation (AFTER) study is a pilot randomised controlled trial to assess feasibility of a later definitive trial comparing best-practice advice with progressive functional exercise for adults aged 50 years and over after ankle fracture.

The main objectives are to assess: (i) patient engagement with the trial, measured by the participation rate of those eligible; (ii) establish whether the interventions are acceptable to participants and therapists, assessed by intervention adherence levels, participant interviews and the therapist focus group; (iii) participant retention in the trial, measured by the proportion of participants providing outcome data at 6 months; (iv) acceptability of measuring outcomes at 3 and 6-month follow-up.

Methods and analysis

A multicentre pilot randomised controlled trial with an embedded qualitative study. At least forty-eight participants aged 50 years and over with an ankle fracture requiring surgical management, or non-operative management by immobilisation for at least 4 weeks, will be recruited from a minimum of three National Health Service hospitals in the United Kingdom. Participants will be allocated 1:1 via a central web-based randomisation system to: i) best-practice advice (one session of face-to-face self-management advice delivered by a physiotherapist and up to two optional additional sessions) or ii) progressive functional exercise (up to six sessions of individual face-to-face physiotherapy). An embedded qualitative study will include one-to-one interviews with up to 20 participants and a therapist focus group.

Ethics and dissemination

Hampshire B Research Ethics Committee (18/SC/0281) gave approval on 2nd July 2018.

Trial registration number

ISRCTN16612336

For peer review only - http://bmjopen.bmj.com/site/about/guidelines.xhtml

Keywords

Ankle Fractures, Clinical Trial, Geriatrics, Exercise Therapy

For peer review only

For peer review only - http://bmjopen.bmj.com/site/about/guidelines.xhtml

Article summary

Strengths and limitations of this study

- The Ankle Fracture Enhancing Rehabilitation (AFTER) study is a pilot randomised controlled trial with an embedded qualitative study recruiting at least 48 patients aged 50 years and over after an ankle fracture from at least three UK National Health Service hospitals.
- Participants will be randomly allocated to either: i) best-practice advice (one session of face-to-face self-management advice delivered by a physiotherapist and up to two more optional advice sessions) or ii) progressive functional exercise (up to six sessions of individual face-to-face physiotherapy).
- The interventions were developed using current research evidence and with input from clinical experts, researchers, and patient and public representatives.
- We aim to assess the feasibility of a future definitive randomised controlled trial in terms of patient engagement with the trial, intervention acceptability and fidelity, retention of participants in the trial, and acceptability of measuring outcomes at 3 and 6-month follow-up.
- Physiotherapists and participants are not blinded due to the nature of the interventions.

For peer review only

INTRODUCTION

Fractures may have a devastating effect on the lower limb, resulting in mobility problems and loss of independence.[1] The incidence of ankle fractures for people aged 50 years and over in the UK is 10.4 per 10,000 person-years.[2] As the proportion of ankle fractures occurring in older adults increases[3 4] and the population ages, a three-fold increase in these injuries by 2030 is projected.[5] A systematic review of outcome data after ankle fracture showed that functional outcomes worsen with increasing age,[6] likely due to lower physiological reserves (frailty), comorbidities, reduced muscle mass and power (sarcopenia),[7] and poor balance.[8] Participants in the Ankle Injury Management (AIM) trial (which recruited participants 60 years and over) self-reported an average 30% loss in ankle function, less confidence in walking, and more fear of falling from pre-injury to 6 months post-injury.[9]

In developed countries, unstable ankle fractures typically undergo internal fixation surgery to restore normal anatomy.[10] Below-knee casts are also used in older adults as they have greater risks from surgery than younger people.[11] The AIM trial showed surgery and close contact casting for managing unstable ankle fractures in older adults had equivalence in functional outcomes.[9] Whatever the initial fracture management, protective splinting (in a cast or boot) to immobilise the ankle joint and support the injured leg is commonplace. Physical impairments after ankle fracture include ankle pain, reduced ankle motion,[12] lower limb muscle strength deficits,[13 14] mobility limitations,[12 15] and walking abnormalities.[16] Many patients see a physiotherapist to aid recovery, usually in outpatient departments as most patients are ambulatory, albeit with difficulty.

A Cochrane review[17] of ankle fracture rehabilitation concluded that there was insufficient evidence to support traditional physiotherapy interventions targeting ankle joint and muscle impairments, such as stretching,[18] manual therapy,[19] and exercise.[20] Updating the Cochrane review searches in MEDLINE and EMBASE, identified another multicentre trial by Moseley and colleagues.[21] They found no differences in self-reported lower limb function or quality of life between supervised exercise and a one-off advice session for adults with ankle fractures treated surgically and conservatively. Physiotherapists delivered both interventions face-to-face. The exercises included traditional ankle exercises, single-limb weight-bearing exercises, and advice on using walking aids. The single advice session intervention had low adherence, with a third of participants obtaining one or more extra sessions of out-of-trial physiotherapy.

For peer review only

Older adults were not adequately represented in either treatment group, as the mean age was 42 years.

Physiotherapy interventions tested to date have not incorporated features of advice and exercise programmes for older adults used in other rehabilitation areas. Issues such as persistent poor balance in older adults require attention.[22] Training for functional movement problems (e.g., walking and stair climbing) and balance, rather than traditional ankle-impairment exercises, has a strong evidence base for older people's rehabilitation. Focusing on ankle impairments in rehabilitation may be insufficient for dealing with complex mobility problems in older adults after injury.[23]

The AFTER study will assess a novel approach to physiotherapy provision, progressive functional exercise. The progressive exercise programme will use contemporary evidence-based guidelines on exercise volume and load to optimise the physiological response. The widely-accepted overload principle states that movement and strength improvements require a training stimulus of sufficient volume and intensity.[24] Strong evidence suggests psychological barriers to adherence to physiotherapy advice and exercises.[25] As participants expressed fear of walking and falling in a qualitative study within the AIM trial, these psychological factors will be addressed.[26]

A pilot randomised controlled trial (RCT) is needed to investigate areas of uncertainty for a future definitive RCT, exploring trial design, recruitment, follow-up, and the acceptability of the interventions and outcome measures.

Objectives

The AFTER (Ankle Fracture Treatment: Enhancing Rehabilitation) study will assess the feasibility of a definitive RCT to compare progressive exercise with best-practice advice in patients aged 50 years or over after ankle fracture.

The main objectives of the pilot trial are to:

- Assess patient engagement with the trial, measured by the participation rate of those eligible.
- Establish whether the interventions are acceptable to participants and therapists, assessed by intervention adherence levels, participant interviews and a therapist focus group.

For peer review only - http://bmjopen.bmj.com/site/about/guidelines.xhtml

- Determine patient retention, measured by the proportion of patients providing outcome data at 6 months
- Assess the acceptability of measuring outcomes at 3 and 6-month follow-up.

METHODS AND ANALYSIS

Study design

A multicentre pilot RCT with an embedded qualitative study. Participants will be allocated to either: i) best-practice advice (one session of face-to-face advice delivered by a physiotherapist, with up to two further optional advice sessions) or ii) progressive functional exercise (up to six sessions of individual face-to-face physiotherapy) (see Figure 1).

Setting

Recruitment will be from at least three NHS hospitals and their related physiotherapy services. Participants will be identified during inpatient stays or when attending fracture clinics, where they will be screened and given study information. Eligibility will be assessed by the treating clinician, usually an orthopaedic surgeon or physiotherapist.

Study participants

The target population is at least 48 adults aged 50 years or over attending NHS services to manage ankle fractures that require definitive management with surgical treatment or ankle immobilisation for at least 4 weeks.

Eligibility

We will include adults aged 50 years or over with an ankle fracture who are undergoing surgical fixation or conservative management involving ankle immobilisation for at least 4 weeks.

Patients will be excluded if they:

- are unable to adhere to trial procedures or complete questionnaires
- who do not have capacity to consent to study participation
- were not ambulatory before the injury
- are considered inappropriate for referral to physiotherapy by the clinician
- cannot attend outpatient physiotherapy at a participating centre

For peer review only - http://bmjopen.bmj.com/site/about/guidelines.xhtml

- have serious concomitant disease (such as terminal illness)
- have bilateral lower limb fractures
- have an ipsilateral concurrent Pilon fracture
- have open fracture wounds, external fixation, or substantial skin loss or grafts that would limit ankle or lower leg exercise

Recruitment

Participants will be recruited from NHS hospitals and related physiotherapy services. Posters displayed in clinics will advertise the AFTER study to patients and clinicians.

Screening and eligibility assessment

Potential participants will be inpatients or attending trauma and orthopaedic clinics. Patients presenting with an ankle fracture will be assessed against the eligibility criteria, given a copy of the participant information sheet, and asked if they wish to be considered for the study. Patients who meet the eligibility criteria and would like to participate will be approached for informed consent. If the potential participant is deemed eligible and is willing to proceed, then they and the researcher will sign and date a consent form.

Patients who do not meet the eligibility criteria or who do not wish to participate will receive standard NHS treatment. We will record the age and gender of those not eligible or who decline participation to assess the generalisability of those recruited. We will ask these patients why they declined the study and record any answers provided.

Randomisation

Consented participants will be randomised 1:1 to the intervention groups using the centralised computer randomisation service provided by the Oxford Clinical Trials Research Unit. The site's research facilitator will undertake randomisation directly themselves or will contact the study office over the telephone to access the system on their behalf. Randomisation will be computer-generated and stratified by centre and initial fracture management (surgery or non-surgical) using a variable block size to ensure participants from each centre have an equal chance of receiving each intervention. Participants will only be randomised after eligibility assessment and informed consent.

For peer review only - http://bmjopen.bmj.com/site/about/guidelines.xhtml

For peer review only

Blinding

Physiotherapists delivering the intervention and study participants will be told the treatment allocation. Researchers independent of the clinical team will collect the objective outcome measures at 6-month follow-up.

Interventions

Training and monitoring of intervention delivery

The best-practice advice and progressive functional exercise interventions will be provided face-to-face and one-to-one by physiotherapists. The study team will give the treating therapists training and an intervention manual on the theory and practical delivery of the interventions. Treating physiotherapists will record the delivery and content of the sessions attended by each participant.

Patient pathways vary at different hospitals so patients may have had instruction on basic ankle exercises, wound care, and use of walking aids from a physiotherapist or other health professional while an inpatient or in an acute fracture clinic before starting the study interventions. This will be recorded in the study treatment log. The intervention session(s) can be tailored to the patient's recovery level, as per usual care. Some centres make referrals for physiotherapy while patients are inpatients or at earlier clinic follow-ups. Other centres delay referrals until a clinic review several weeks later. The study will adapt screening and recruitment to embed in the patient pathway as much as is practical. The process at each centre will be reviewed as the study progresses.

The best-practice advice or progressive functional exercise sessions will be given when the participant can mobilise with unrestricted weight-bearing and do ankle exercises as guided by their surgeon or physiotherapist. We anticipate that this will usually be around 6-8 weeks post-injury.

Best-practice advice

A single advice session with a physiotherapist with one or two follow-up appointments is commonplace in the UK NHS. This was considered an acceptable provision model reflecting best-practice usual care advice in a consensus meeting with health care professionals and patient and public involvement representatives held to help design the AFTER study. There are no clinical guidelines on physiotherapy for ankle fractures. Recent NICE fracture

For peer review only - http://bmjopen.bmj.com/site/about/guidelines.xhtml

guidelines made no specific recommendations for ankle fracture physiotherapy, but did highlight the importance of supporting advice with written information to enhance self-management.[27]

There is variation in physiotherapy provision in the NHS. However, as with many physiotherapy trials, the volume and content will be standardised to manage heterogeneity and ensure a reproducible intervention that can be assessed for generalisability. This intervention should be a credible representation of current best-practice usual care advice across the NHS. Key stakeholders, including clinical experts, researchers, and patient and public representatives, contributed substantially to the development of the interventions during the design phase of the AFTER study.

The best-practice advice intervention will focus on self-management. It will include a 20- to 60-minute session (depending on local service provision) of assessment, education, reassurance, and detailed self-management advice on ankle exercises, gait training, stair climbing, walking aid advice, and basic balance exercises. An advice booklet will provide key information.

Up to two further sessions advice will be optional for participants that are having difficulties with self-management or the exercises. The physiotherapist's role will be to re-assess and re-enforce self-management advice. During the initial stage of recruitment these additional sessions may involve a telephone call or an additional face-to-face contact. In the latter stage of recruitment, these sessions will be offered as telephone consultations only. Use of additional sessions will be recorded and monitored.

Physiotherapists use a range of passive manual and electrotherapy modalities but clinical trials and systematic reviews have found limited evidence that these treatments effectively improve outcomes after ankle fracture.[17] They will therefore not be a core part of the best-practice advice but use will be recorded in the treatment logs.

Progressive functional exercise

Participants will undertake progressive functional resistance training and balance exercises. A physiotherapist will instruct on, supervise, and progress the functional exercises in up to six sessions over 16 weeks. This period allows sufficient time for neuromuscular adaptation to exercise.[28] The programme can end early if all rehabilitation goals are achieved in under six sessions. The first session will be 20-60 minutes and the rest up to 30 minutes, consistent

For peer review only
For peer review only - http://bmjopen.bmj.com/site/about/guidelines.xhtml

with physiotherapy sessions in the NHS. Physiotherapists will provide assessment, advice, education about progressing recovery, gait training, walking aid instruction, and an information booklet.

The programme will be highly structured but calibrated for each individual. Tailoring programmes to the participant is a key feature of effective interventions in older adults.[29] All participants will receive a core set of functional lower limb strengthening exercises in line with the evidence for improving muscular strength and power in older persons' rehabilitation.[30] Strength improvements do not necessarily translate to function and balance improvements. The programme will include supervised gait training to target major walking difficulties after ankle fracture. Gait training improves motor control during walking in older adults, which is related to functional mobility and balance improvements.[31] Balance exercises will also be included in the programme and introduced once the participant is able to weight bear sufficiently to perform these. Exercises will be practised in the clinic but conducted at home to achieve an effective dose. Based on the participant's functional goals, exercises will be progressed to make them task-specific, for example walking on uneven surfaces or slopes, climbing stairs, or jogging. The participants will receive a personal exercise guide and diary. Exercise progression will be based on evidence-based guidelines[24] but individualised by progressing and regressing the volume and load in line with each participant's capabilities and preferences.

The progressive functional exercise intervention will use simple health behaviour change techniques to optimise adherence to home exercise. We drew on the evidence-based NHS Health Trainers Handbook, recommended for routine use by health professionals to promote patient behaviour change.[32] Our group has used these techniques in other physiotherapy trials to develop feasible exercise programmes that have resulted in self-efficacy improvements.[33] The strategies use a two-stage mechanism, increasing intention to adhere to the exercise regimen and translating this behavioural intention into actual behaviour.

Participants will be asked to identify their goals following usual physiotherapy practice and, with the treating therapist's help, write an action plan for where and when they will perform their home exercises and a contingency plan for managing difficulties.

Therapists will be trained to focus on helping participants identify barriers to exercise and becoming more physically active post-injury, and facilitating problem-solving. The therapists will offer education on how exercise and physical activity can help participants to achieve

For peer review only - http://bmjopen.bmj.com/site/about/guidelines.xhtml

their goals and will reassure participants about their capacity to exercise and increase their physical activity.[34] The intervention will give participants individualised feedback on their rehabilitation progress and reinforcement over the sessions, and will facilitate identification of barriers to doing the home exercise programme, which all have a strong evidence base to support their use in older adults.[29]

There is evidence that patients do not retain much of the information received face-to-face.[35] This sense of being overwhelmed by verbal instructions from physiotherapists was echoed by several patient and public involvement (PPI) group representatives, who strongly advocated quality supporting materials. A high-quality information booklet will be developed by the AFTER study team and provided to participants.

Concomitant care

Other aspects of health and social care will continue as normal. Analgesia use will be self-reported. Participants may seek other forms of treatment during follow-up, but will be asked to use their usual routes of access or referral to do so. Additional treatments, including contact with their GP or other health professionals, will be recorded in participant follow-up questionnaires.

A rigorous quality control programme will ensure intervention fidelity.[36] Participants can seek care outside the study, which will be recorded as part of health resource use. Participant crossover between intervention groups will not be allowed. The local site coordinating physiotherapist and study team will share responsibility for intervention quality control. Site visits will be conducted periodically to observe recruitment, consent, randomisation, data collection, and progressive exercise and best-practice advice sessions. Permission will be sought from the participants to observe treatment sessions. Data will be collected on intervention delivery, number of treatment sessions attended, and details about the core and adaptable components to facilitate monitoring and reporting. Sites will regularly receive feedback from quality control visits to help maintain and improve fidelity. Identified issues will be addressed by engaging the site staff in more training and increasing monitoring by the central trial team.

Outcome measures

Feasibility criteria

For peer review only - http://bmjopen.bmj.com/site/about/guidelines.xhtml

The main aim of this pilot RCT is to determine the feasibility of a future definitive trial.[37] The focus will not be on a primary outcome of effectiveness, but instead on meeting success criteria. The main uncertainty is whether patients find it acceptable to be randomised to different types of physiotherapy. Screening data from the AIM trial gives confidence that there are enough potential participants to investigate the feasibility criteria. To determine the feasibility of a definitive RCT, the success criteria are:

- A study participation rate of at least 25% of those eligible, to indicate acceptability and generalisability.
- At least 48 eligible participants across at least three sites agree to participate over a maximum of 18 months
- At least 85% of participants complete the study intervention sessions
- At least 80% of participants attend study follow-up at 6 months

Descriptions from the participant interviews and therapist focus group that indicate randomisation/interventions are acceptable will also be used to assess feasibility. Treatment logs will monitor intervention fidelity and tolerability. Adherence to home exercise will be monitored via participant self-reports. An estimate of standard deviation for the primary outcome is not required as the AIM trial will provide this information for the definitive trial.

Outcomes

Outcomes will be collected to assess the feasibility of their collection in a future definitive RCT (see Tables 1 and 2).

Patient-reported outcomes at 3 and 6 months will be:

- ankle-related symptoms and function: Olerud and Molander Ankle Score[38]
- lower-limb function limitations: Lower Extremity Functional Scale[39]
- pain: visual analogue scale (VAS), 0-100 scale
- health-related quality of life: EQ-5D-5L score[40]
- fear of falls: Falls Efficacy Scale-International (short version)[41]
- self-efficacy: self-efficacy exercise score[42]
- return to desired activities, including work, social life, and sport activities
- walking aid use and distances
- exercise adherence

For peer review only - http://bmjopen.bmj.com/site/about/guidelines.xhtml

At 6-month follow-up, a blinded outcome assessor will collect objective measures of ankle function and physical performance:

- ankle joint range: hand-held goniometry[43]
- muscle strength: hand-held dynamometry (Lafayette Manual Muscle Test System, Lafayette Instrument, IN, USA) of ankle dorsi/plantarflexion using a 'make' approach (working up to a maximal contraction over a maximum of 5 seconds and without pushing into pain and the assessor maintaining position of the device).[44] Participants will be measured 3 times and have at least 10 seconds rest between attempts. Self-reported body weight will be recorded to aid interpretation of strength measures.
- mobility and balance: Short Physical Performance Battery (SPPB).[45] The test involves physical tests of balance, walking speed, and repeated rises from a chair. It has been used extensively in clinical trials owing to its practical utility, the strong evidence base for its measurement properties, and its relationship to frailty, risk of falls, and disability in older adults.[46]

Data on health resource use (consultation with primary and secondary care, prescribed and over-the-counter medication use, additional physiotherapy, and hospital admission), additional out-of-pocket expenses, and work absence (number of sickness days) will also be collected to inform a future definitive RCT. A full health economic evaluation will not be conducted. Data will be collected alongside the other outcome measures in participant questionnaires.

Adverse events

Foreseeable adverse events (AE) occurring as a result of the trial intervention(s) will be recorded. Participants will receive information on the potential AEs resulting from the treatment exercises and what they should do if they experience an AE, as would happen as part of standard NHS procedures.

Expected general side effects of any exercise, such as delayed-onset muscle soreness and temporary increases in pain <1 week, will not be recorded as AEs. Pain increases >1 week will be recorded in patient-reported questionnaires. Although unlikely, any exacerbations of other medical conditions during exercise or exercise-related injurious falls will be recorded in patient-reported questionnaires or by the site investigators if they become aware of such an event.

For peer review only - http://bmjopen.bmj.com/site/about/guidelines.xhtml

A serious AE (SAE) is any unexpected untoward medical occurrence related to the trial interventions that results in death, is life-threatening, requires inpatient hospitalisation or prolongation of existing hospitalisation, or results in persistent or significant disability/incapacity. SAEs are likely to be rare and are unlikely to occur as a result of the exercise programmes delivered in this study. If an SAE arises between study enrolment and final follow-up visit and is deemed related to the trial interventions, the Clinical Trials Unit standard operating procedures will apply.

Follow-up data collection

Participants will be followed-up 3 months after randomisation with a postal questionnaire and 6 months after randomisation face-to-face at the hospital. They will be offered telephone, postal, or electronic follow-up if they are unable to attend the 6-month follow-up.

Participants will be asked to complete the 3-month (and, if applicable, 6-month) questionnaire(s) and return it to the AFTER trial team. Those who do not respond to the initial questionnaire will be sent at least one reminder by telephone, SMS text messaging, or email. Telephone and electronic follow-up will also be used to collect a core set of questionnaire items if these are not completed on the returned questionnaire.

Sample size

The main feasibility objective and therefore the basis of the sample size estimate is participant recruitment at three centres with a staggered start. The target sample size is a minimum of 48 participants. Based on three sites and staggered starts, this is equivalent to at least 1.5 participants per month per site over 12 months. This sample size will enable an estimate of a minimum 25% recruitment rate of those eligible to within a 95% confidence interval of ±6% (calculated using the modified Wald method).[47] If the sample size is achieved sooner than 12 months, recruitment will continue up to a maximum of 60 participants to enable further feasibility assessment regarding the change to the best practice advice intervention. Recruitment will cease if the target minimum sample size is not achieved within 18 months. We have selected a conservative recruitment rate as recruitment to previous ankle fracture exercise trials in younger adults with less complex health needs has been 37-80%.[20 21]

Statistical analysis

For peer review only - http://bmjopen.bmj.com/site/about/guidelines.xhtml

Feasibility outcomes will be reported, including the number of participants who are approached, are eligible, consent to randomisation, and are followed-up, attendance of intervention sessions, completion rates of exercise diaries, and data completeness. Baseline characteristics will be reported using descriptive statistics, per group and overall, using mean and standard deviation (or median and interquartile range if non-normally distributed), and minimum and maximum, for continuous variables and number and percentage of patients in each group for binary or categorical variables.

Clinical outcome measures will be reported descriptively. Differences between treatments for the intention-to-treat population will be reported with 95% confidence intervals. Withdrawals from treatment and the trial will be reported, with reasons where provided. AEs, and SAEs will be reported, both the number of participants that experience an event and the total number.

The primary statistical analysis will be carried out on the basis of intention-to-treat, with all randomised participants included and analysed according to their allocated treatment group, irrespective of which treatment they actually receive or their treatment compliance.

Embedded qualitative study

Investigating patient experience is fundamental to understanding how interventions affect patients' lives and will give insights into the acceptability of the trial interventions and randomisation to them. The embedded qualitative study aims to find out more about the patients' experience of the two interventions within the context of their recovery from ankle fracture. This understanding will help us review the acceptability of the two interventions, which aspects help or hinder recovery. This will enable us to refine the interventions, retaining aspects that are important to patients and developing or removing those that are less helpful. For instance, patients may struggle with the pace or complexity of the progressive exercises, which could then be modified for the future definitive trial. In addition the interviews will provide valuable insight into how patients experience the trial processes. For example we know that patients take part in studies because they place their trust in the clinical team, they can have trouble understanding some information, can find randomisation unacceptable in clinical situations and have therapeutic misconceptions.[48-50] Gaining an insight into the aspects that facilitate and limit participation in the study will help us refine our information processes to include areas that are of concern to patients.

For peer review only - http://bmjopen.bmj.com/site/about/guidelines.xhtml

Interviews will be undertaken with a purposive sample of up to 20 participants from recruiting centres and each intervention group, approximately 4 months after randomisation. Participants will be invited to take part and provide their agreement to be contacted for an interview after they have consented to the main pilot trial. Written informed consent will be provided prior to the interview. The interviews will provide an insight into patient experience of being in the trial, the interventions, and recovery and outcomes that are important to them within the context of their life. Factors that inhibit or facilitate their ability to fully take part in the study will be used to inform a large-scale definitive RCT.

To sensitise the research team to factors that are important to study participants the semi-structured interview schedule will be informed by input from a PPI member and a therapist. Open questions will be used to ensure participants can talk freely about what is important to them. Participants will choose whether to be interviewed in the local hospital, over the telephone, or at home.

The experiences of therapists delivering the trial interventions and recruiting patients will also be explored. Therapists will be invited to participate in a focus group to be held at the end of the recruitment phase.

Interviews and the focus group will be audio-recorded, transcribed verbatim and NVivo (QSR International Pty Ltd, Melbourne, Australia) will be used to help manage the data. Data will be coded and grouped into categories and themes drawing on thematic analysis.[51] Any quotes used in reporting of the findings will be anonymised.

PATIENT AND PUBLIC INVOLVEMENT

The development of the study funding application, intervention development and study materials were supported by a patient and public involvement group, who will also be involved in developing the dissemination strategy.

ETHICS AND DISSEMINATION

The study has been approved by the South Central – Hampshire B Research Ethics Committee (Ref: 18/SC/0281) and the Health Research Authority. It has been prospectively registered (ISRCTN16612336). All protocol amendments are will be subject to review by the

For peer review only - http://bmjopen.bmj.com/site/about/guidelines.xhtml

Sponsor (Oxford University) the ethics committee and Health Research Authority and will be included in the final report. All data will be processed following Oxford Clinical Trials Research Unit (OCTRU) standard operating procedures. Adverse events and serious adverse events are likely to be rare. If they occur they will be reviewed by the trial management group.

This protocol has been reported following the Standard Protocol Items: Recommendations for Interventional Trials (SPIRIT) statement.[52] Results will be published in an open-access journal, reported following the Consolidated Standards of Reporting Trials (CONSORT) guidelines for pilot and feasibility trials.[53 54] The Template for Intervention Description and Replication (TIDieR) statement will be used to report the intervention,[55] ensuring replication is possible. Results will be published in a peer-reviewed journal with authorship eligibility according to the ICMJE criteria. Participants will be asked if and how they would like to be informed of the study results during the consent process. We will share study results before publication with those participants who request it.

For peer review only

For peer review only - http://bmjopen.bmj.com/site/about/guidelines.xhtml

Acknowledgements

The AFTER study collaborating Principal Investigators are:

Hannah Perkins (Oxford University Hospitals NHS Foundation Trust)

Carol McCrum (East Sussex Healthcare NHS Trust)

Jacky Jones (Guy's and St Thomas' NHS Foundation Trust)

Carey McClellan (University Hospitals NHS Foundation Trust)

Fiona Cowell (Royal Liverpool and Broadgreen University Hospital NHS Trust)

We acknowledge English language editing by Dr Jennifer A de Beyer of the Centre for Statistics in Medicine (University of Oxford).

Author contributions

DJK is the chief investigator and conceived and designed the study, was awarded the funding, , and drafted the manuscript. MLC, ET, SH, VSB, SJD, ACR, KW and SEL contributed to study design and provided specific content and edited the manuscript. SJD oversees the statistical aspects of the study. ET oversees the qualitative sub-study. VSB has provided trial management oversight. All authors have reviewed and approved the manuscript.

Funding statement

This report is independent research supported by the National Institute for Health Research (NIHR Post-Doctoral Fellowship, Dr David Keene, PDF-2016-09-056). The report was supported by the NIHR Biomedical Research Centre, Oxford. Professor Lamb receives funding from the NIHR Collaboration for Leadership in Applied Health Research and Care Oxford at Oxford Health NHS Foundation Trust.

Disclaimer

The views expressed in this publication are those of the author(s) and not necessarily those of the NHS, the National Institute for Health Research, or the Department of Health.

The study sponsor and funders had no role in study design; writing of the report; and the decision to submit the report for publication.

Data statement

For peer review only

Data sharing request procedures will be reported in the published results manuscript.

Competing interests statement

None declared.

For peer review only

References

1. Pasco JA, Sanders KM, Hoekstra FM, et al. The human cost of fracture. *Osteoporos Int* 2005;16(12):2046-52.
2. Curtis EM, van der Velde R, Moon RJ, et al. Epidemiology of Fractures in the United Kingdom 1988-2012: Variation with age, sex, geography, ethnicity and socioeconomic status. *Bone* 2016;87:19-26. doi: 10.1016/j.bone.2016.03.006
3. Thur CK, Edgren G, Jansson KA, et al. Epidemiology of adult ankle fractures in Sweden between 1987 and 2004: A population-based study of 91,410 Swedish inpatients. *Acta Orthop* 2012;83(3):276-81. doi: 10.3109/17453674.2012.672091
4. Court-Brown CM, Caesar B. Epidemiology of adult fractures: A review. *Injury* 2006;37(8):691-7. doi: 10.1016/j.injury.2006.04.130
5. Kannus P, Palvanen M, Niemi S, et al. Stabilizing incidence of low-trauma ankle fractures in elderly people Finnish statistics in 1970-2006 and prediction for the future. *Bone* 2008;43(2):340-42. doi: S8756-3282(08)00225-1 [pii]

10.1016/j.bone.2008.04.015

6. Beckenkamp PR, Lin CW, Chagpar S, et al. Prognosis of physical function following ankle fracture: a systematic review with meta-analysis. *J Orthop Sports Phys Ther* 2014;44(11):841-51, B2. doi: 10.2519/jospt.2014.5199
7. Clegg A, Young J, Iliffe S, et al. Frailty in elderly people. *Lancet* 2013;381(9868):752-62. doi: 10.1016/S0140-6736(12)62167-9
8. Seeley DG, Kelsey J, Jergas M, et al. Predictors of ankle and foot fractures in older women. The Study of Osteoporotic Fractures Research Group. *J Bone Miner Res* 1996;11(9):1347-55. doi: 10.1002/jbmr.5650110920
9. Keene DJ, Mistry D, Nam J, et al. The Ankle Injury Management (AIM) Trial: A pragmatic, multi-centre, equivalence randomised controlled trial and economic evaluation comparing close contact casting with open surgical reduction and internal fixation in the treatment of unstable ankle fractures in patients over 60 years. *Health Technol Assess*;In press
10. Handley R, Gandhe A. Ankle fractures. In: Bulstrode CJK, ed. Oxford textbook of trauma and orthopaedics. 2nd ed. Oxford: Oxford University Press 2011.
11. Willett K, Keene DJ, Morgan L, et al. Ankle Injury Management (AIM): design of a pragmatic multi-centre equivalence randomised controlled trial comparing Close Contact Casting (CCC) to Open surgical Reduction and Internal Fixation (ORIF) in the treatment of unstable ankle fractures in patients over 60 years. *BMC Musculoskelet Disord* 2014;15(1):79. doi: 10.1186/1471-2474-15-79
12. Lin CW, Moseley AM, Herbert RD, et al. Pain and dorsiflexion range of motion predict short- and medium-term activity limitation in people receiving physiotherapy intervention after ankle fracture: an observational study. *Aust J Physiother* 2009;55(1):31-7.
13. Stevens JE, Walter GA, Okereke E, et al. Muscle adaptations with immobilization and rehabilitation after ankle fracture. *Med Sci Sports Exerc* 2004;36(10):1695-701.
14. Psatha M, Wu Z, Gammie FM, et al. A longitudinal MRI study of muscle atrophy during lower leg immobilization following ankle fracture. *J Magn Reson Imaging* 2012;35(3):686-95. doi: 10.1002/jmri.22864
15. Hancock MJ, Herbert RD, Stewart M. Prediction of outcome after ankle fracture. *J Orthop Sports Phys Ther* 2005;35(12):786-92.
16. Wolf SI, Lessl E, Müller S, et al. Persistent diminished joint function following ankle fracture - a risk factor for posttraumatic osteoarthritis? [abstract]. *Gait Posture* 2013;38 (Suppl 1):S101-02. doi: http://dx.doi.org/10.1016/j.gaitpost.2013.07.207

For peer review only - http://bmjopen.bmj.com/site/about/guidelines.xhtml

17. Lin CW, Donkers NA, Refshauge KM, et al. Rehabilitation for ankle fractures in adults. *Cochrane Database Syst Rev* 2012;11:Cd005595. doi: 10.1002/14651858.CD005595.pub3
18. Moseley AM, Herbert RD, Nightingale EJ, et al. Passive stretching does not enhance outcomes in patients with plantarflexion contracture after cast immobilization for ankle fracture: a randomized controlled trial. *Arch Phys Med Rehabil* 2005;86(6):1118-26. doi: 10.1016/j.apmr.2004.11.017
19. Lin CW, Moseley AM, Haas M, et al. Manual therapy in addition to physiotherapy does not improve clinical or economic outcomes after ankle fracture. *J Rehabil Med* 2008;40(6):433-9. doi: 10.2340/16501977-0187
20. Nilsson GM, Jonsson K, Ekdahl CS, et al. Effects of a training program after surgically treated ankle fracture: a prospective randomised controlled trial. *BMC Musculoskelet Disord* 2009;10:118. doi: 10.1186/1471-2474-10-118
21. Moseley AM, Beckenkamp PR, Haas M, et al. Rehabilitation After Immobilization for Ankle Fracture: The EXACT Randomized Clinical Trial. *JAMA* 2015;314(13):1376-85. doi: 10.1001/jama.2015.12180
22. Dudek K, Druzbicki M, Przysada G, et al. Assessment of standing balance in patients after ankle fractures. *Acta Bioeng Biomech* 2014;16(4):59-65.
23. Ebrahim S, Adamson J, Ayis S, et al. Locomotor disability: Meaning, causes and effects of interventions. *J Health Serv Res Policy* 2008;13 Suppl 3:38-46. doi: 13/suppl_3/38 [pii]

10.1258/jhsrp.2008.008013
24. Kraemer W, Adams K, Cafarelli E, et al. American College of Sports Medicine position stand. Progression models in resistance training for healthy adults. *Med Sci Sports Exerc* 2002;34(2):364-80.
25. Jack K, McLean SM, Moffett JK, et al. Barriers to treatment adherence in physiotherapy outpatient clinics: a systematic review. *Man Ther* 2010;15(3):220-8. doi: 10.1016/j.math.2009.12.004
26. Ayis S, Gooberman-Hill R, Bowling A, et al. Predicting catastrophic decline in mobility among older people. *Age Ageing* 2006;35(4):382-87. doi: 10.1093/ageing/afl004
27. National Institute for Clinical and Care Excellence. Draft for consultation: Fractures (non-complex): assessment and management: National Clinical Guideline Centre 2015.
28. Byrne C, Faure C, Keene DJ, et al. Ageing, muscle power, and physical function: A systematic review and implications for pragmatic training interventions. *Sports Med* 2016;In Press
29. Zubala A, MacGillivray S, Frost H, et al. Promotion of physical activity interventions for community dwelling older adults: A systematic review of reviews. *PLoS One* 2017;12(7):e0180902. doi: 10.1371/journal.pone.0180902
30. National Institute for Clinical and Care Excellence. Falls in older people: Assessing risk and prevention. Manchester: NICE 2013.
31. Brach JS, Lowry K, Perera S, et al. Improving motor control in walking: A randomized clinical trial in older adults with subclinical walking difficulty. *Arch Phys Med Rehabil* 2015;96(3):388-94. doi: 10.1016/j.apmr.2014.10.018
32. Michie S, Rumsey N, Fussell A, et al. Improving health: Changing behaviour.: NHS health trainer handbook, 2008.
33. Lamb SE, Williamson EM, Heine PJ, et al. Exercises to improve function of the rheumatoid hand (SARAH): A randomised controlled trial. *Lancet* 2015;385(9966):421-29. doi: 10.1016/S0140-6736(14)60998-3
34. Dobson F, Bennell KL, French SD, et al. Barriers and Facilitators to Exercise Participation in People with Hip and/or Knee Osteoarthritis: Synthesis of the Literature Using

For peer review only - http://bmjopen.bmj.com/site/about/guidelines.xhtml

Behavior Change Theory. *Am J Phys Med Rehabil* 2016;95(5):372-89. doi: 10.1097/phm.0000000000000448
35. Kessels RPC. Patients' memory for medical information. *J R Soc Med* 2003;96(5):219-22.
36. Hasson H. Invervention fidelity in clinical trials. In: Richards DA, Hallberg IR, eds. Complex interventions in health. Abingdon: Routledge 2015.
37. Thabane L, Ma J, Chu R, et al. A tutorial on pilot studies: The what, why and how. *BMC Med Res Methodol* 2010;10:1. doi: 10.1186/1471-2288-10-1
38. Olerud C, Molander H. A scoring scale for symptom evaluation after ankle fracture. *Arch Orthop Trauma Surg* 1984;103(3):190-4.
39. Binkley JM, Stratford PW, Lott SA, et al. The Lower Extremity Functional Scale (LEFS): Scale Development, Measurement Properties, and Clinical Application. *Phys Ther* 1999;79(4):371-83. doi: 10.1093/ptj/79.4.371
40. Brooks R. EuroQol: the current state of play. *Health Policy* 1996;37(1):53-72.
41. Kempen GIJM, Yardley L, Van Haastregt JCM, et al. The Short FES-I: a shortened version of the falls efficacy scale-international to assess fear of falling. *Age and Ageing* 2008;37(1):45-50. doi: 10.1093/ageing/afm157
42. Resnick B, Jenkins LS. Testing the Reliability and Validity of the Self-Efficacy for Exercise Scale. *Nurs Res* 2000;49(3):154-59.
43. Bennell KL, Talbot RC, Wajswelner H, et al. Intra-rater and inter-rater reliability of a weight-bearing lunge measure of ankle dorsiflexion. *Aust J Physiother* 1998;44(3):175-80.
44. Wang C-Y, Olson SL, Protas EJ. Test-retest strength reliability: hand-held dynamometry in community-dwelling elderly fallers. *Arch Phys Med Rehabil* 2002;83(6):811-15.
45. Guralnik JM, Simonsick EM, Ferrucci L, et al. A short physical performance battery assessing lower extremity function: Association with self-reported disability and prediction of mortality and nursing home admission. *J Gerontol* 1994;49(2):M85-M94. doi: 10.1093/geronj/49.2.M85
46. Freiberger E, de Vreede P, Schoene D, et al. Performance-based physical function in older community-dwelling persons: A systematic review of instruments. *Age Ageing* 2012;41(6):712-21. doi: 10.1093/ageing/afs099
47. Agresti A, Coull BA. Approximate is better than "exact" for interval estimation of binomial proportions. *Am Stat* 1998;52(2):119-26. doi: 10.1080/00031305.1998.10480550
48. Keene DJ, Mistry D, Nam J, et al. The Ankle Injury Management (AIM) trial: a pragmatic, multicentre, equivalence randomised controlled trial and economic evaluation comparing close contact casting with open surgical reduction and internal fixation in the treatment of unstable ankle fractures in patients aged over 60 years. *Health Technol Assess* 2016;20(75):1-158. doi: 10.3310/hta20750
49. Tutton E, Achten J, Lamb SE, et al. A qualitative study of patient experience of an open fracture of the lower limb during acute care. *Bone Joint J* 2018;100-B(4):522-26. doi: 10.1302/0301-620X.100B4.BJJ-2017-0891.R1
50. Phelps EE, Tutton E, Griffin X, et al. A qualitative study of patients' experience of recovery after a distal femoral fracture. *Injury* 2019 doi: 10.1016/j.injury.2019.07.021
51. Braun V, Clarke V. Using thematic analysis in psychology. *Qual Res Psychol* 2006;3(2):77-101. doi: 10.1191/1478088706qp063oa
52. Chan AW, Tetzlaff JM, Gotzsche PC, et al. SPIRIT 2013 explanation and elaboration: guidance for protocols of clinical trials. *BMJ* 2013;346:e7586. doi: 10.1136/bmj.e7586
53. Schulz KF, Altman DG, Moher D. CONSORT 2010 statement: updated guidelines for reporting parallel group randomised trials. *BMJ* 2010;340:c332. doi: 10.1136/bmj.c332
54. Eldridge SM, Chan CL, Campbell MJ, et al. CONSORT 2010 statement: extension to randomised pilot and feasibility trials. *BMJ* 2016;355:i5239. doi: 10.1136/bmj.i5239

For peer review only - http://bmjopen.bmj.com/site/about/guidelines.xhtml

55. Hoffmann TC, Glasziou PP, Boutron I, et al. Better reporting of interventions: template for intervention description and replication (TIDieR) checklist and guide. *Bmj* 2014;348:g1687. doi: 10.1136/bmj.g1687

For peer review only

Table 1: Time points at which the outcomes will be assessed

TIMEPOINT	Enrolment	Allocation	0-4 Months*	3-month follow up*	6-month follow up
Screening log	✓				
Informed consent	✓				
Eligibility confirmed	✓				
Randomisation		✓			
Control: best practice advice			1 physio session (up to 2 more if struggling)		
Intervention: Progressive functional exercise			Up to 6 physio sessions		
Baseline questionnaire	✓				
Follow-up questionnaire				✓	✓
Follow-up clinic visit at hospital					✓
Follow-up reminders				✓	✓
Qualitative interview (optional)			✓		

*The first three month follow-up occurs while participants are doing the recommended exercises, and for the progressive exercise group, they may still be returning for sessions with the physiotherapist.

For peer review only

Table 2: Participant timeline

Outcome	Measurement	Time point
Demographic	age, gender, height, weight, ethnicity, smoking, alcohol consumption, date of injury, fracture classification, initial fracture management, pre-injury walking aid use and exercise tolerance, current work status, level of education, place of residence, social support	Baseline
Ankle-related symptoms and function	Olerud and Molander Ankle Score[38]	Baseline, 3 and 6 months
Lower limb function limitations	Lower Extremity Functional Scale	Baseline, 3 and 6 months
Pain	Visual Analogue Scale (VAS)	Baseline, 3 and 6 months
Health-related quality of life	EQ-5D-5L score[40]	Baseline, 3 and 6 months
Fear of falls	Falls Efficacy Scale-International (short)	Baseline, 3 and 6 months
Self-efficacy	Self-efficacy exercise score[42]	3 and 6 months
Return to desired activities, including work, social life and sport activities	Patient-reported return to activities	3 and 6 months
Walking aid use and distance	Patient-reported use of aids and maximum distance walked on any single occasion	Baseline (recall pre-injury and current status), 3 and 6 months
Adherence to exercise	Patient-reported exercise performance	3 and 6 months
Mobility and balance	Short Physical Performance Battery (SPPB)[45]	6 months
Ankle joint range	Hand-held goniometry[43]	6 months
Muscle strength	Hand-held dynamometry	6 months
Medication usage	Prescribed and over-the-counter medications	Baseline, 3 and 6 months
Work disability	Sick leave (days)	Baseline, 3 and 6 months
Healthcare use	NHS outpatient and community services (e.g., GP, additional physical therapy)<br>NHS inpatient and day case (e.g., radiography, readmissions)	3 and 6 months

For peer review only - http://bmjopen.bmj.com/site/about/guidelines.xhtml

Outcome	Measurement	Time point
	Private health care services	
Out-of-pocket expenses	Patient-related out-of-pocket expenses recording form	3 and 6 months
Adverse events	Patient-reported adverse events	3 and 6 months

For peer review only

Figure 1: Study flow diagram for the AFTER study

For peer review only

For peer review only - http://bmjopen.bmj.com/site/about/guidelines.xhtml

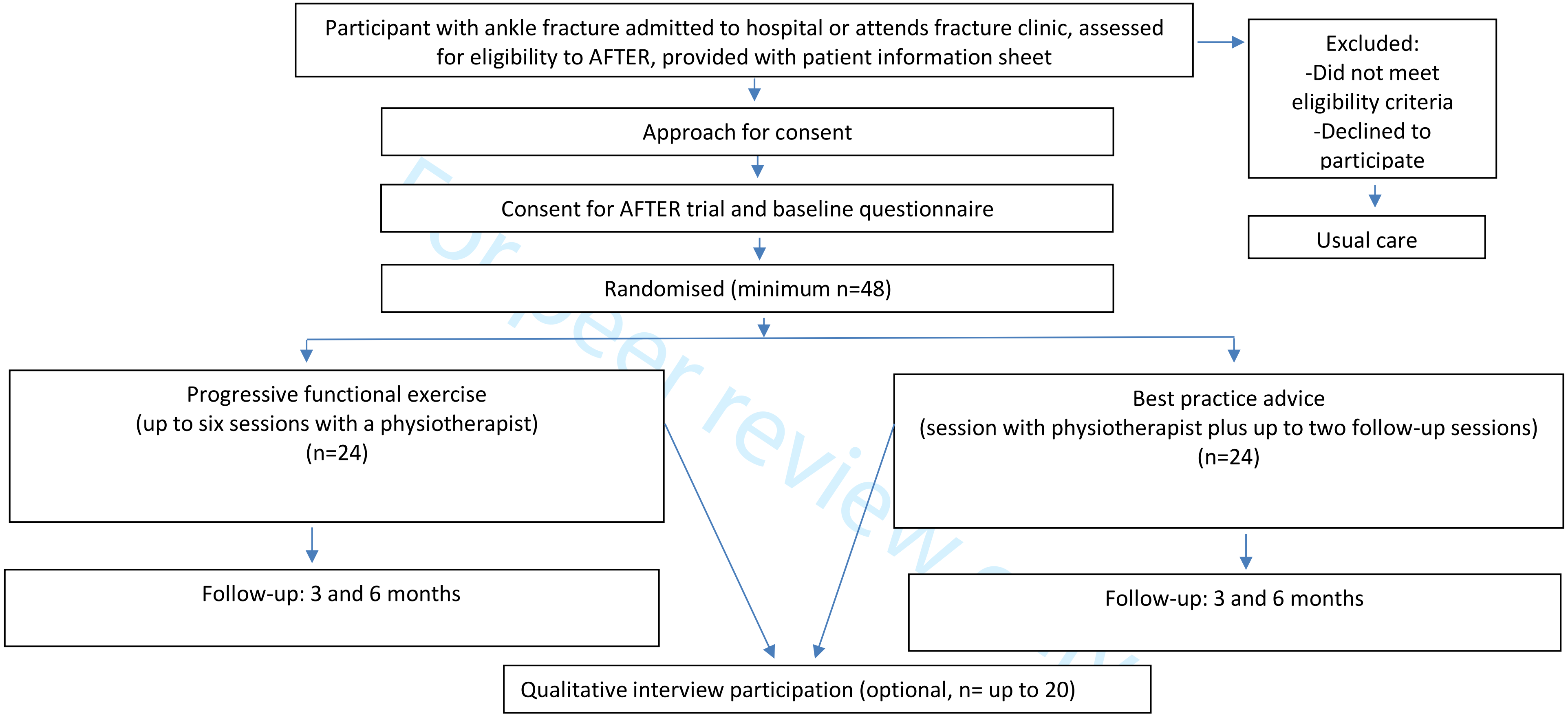

For peer review only - http://bmjopen.bmj.com/site/about/guidelines.xhtml

STANDARD PROTOCOL ITEMS: RECOMMENDATIONS FOR INTERVENTIONAL TRIALS

SPIRIT 2013 Checklist: Recommended items to address in a clinical trial protocol and related documents*

Section/item	ItemNo	Description	Reported
**Administrative information**			
Title	1	Descriptive title identifying the study design, population, interventions, and, if applicable, trial acronym	p.1
Trial registration	2a	Trial identifier and registry name. If not yet registered, name of intended registry	p.2
	2b	All items from the World Health Organization Trial Registration Data Set	p.2 linked registration
Protocol version	3	Date and version identifier	Publication is based on final version
Funding	4	Sources and types of financial, material, and other support	p.19
Roles and responsibilities	5a	Names, affiliations, and roles of protocol contributors	p.1 and 19
	5b	Name and contact information for the trial sponsor	p.17 and 18

For peer review only

	5c	Role of study sponsor and funders, if any, in study design; collection, management, analysis, and interpretation of data; writing of the report; and the decision to submit the report for publication, including whether they will have ultimate authority over any of these activities	p.19
	5d	Composition, roles, and responsibilities of the coordinating centre, steering committee, endpoint adjudication committee, data management team, and other individuals or groups overseeing the trial, if applicable (see Item 21a for data monitoring committee)	p.14 and 15
**Introduction**			
Background and rationale	6a	Description of research question and justification for undertaking the trial, including summary of relevant studies (published and unpublished) examining benefits and harms for each intervention	p.5-6
	6b	Explanation for choice of comparators	p.5-6
Objectives	7	Specific objectives or hypotheses	p.6 to 7

For peer review only

Trial design	8	Description of trial design including type of trial (eg, parallel group, crossover, factorial, single group), allocation ratio, and framework (eg, superiority, equivalence, noninferiority, exploratory)	p.7

Methods: Participants, interventions, and outcomes

Study setting	9	Description of study settings (eg, community clinic, academic hospital) and list of countries where data will be collected. Reference to where list of study sites can be obtained	p.7
Eligibility criteria	10	Inclusion and exclusion criteria for participants. If applicable, eligibility criteria for study centres and individuals who will perform the interventions (eg, surgeons, psychotherapists)	p.7-8
Interventions	11a	Interventions for each group with sufficient detail to allow replication, including how and when they will be administered	p.9-12

For peer review only - http://bmjopen.bmj.com/site/about/guidelines.xhtml

	11b	Criteria for discontinuing or modifying allocated interventions for a given trial participant (eg, drug dose change in response to harms, participant request, or improving/worsening disease)	p.10 and 12
	11c	Strategies to improve adherence to intervention protocols, and any procedures for monitoring adherence (eg, drug tablet return, laboratory tests)	p.12
	11d	Relevant concomitant care and interventions that are permitted or prohibited during the trial	Y
Outcomes	12	Primary, secondary, and other outcomes, including the specific measurement variable (eg, systolic blood pressure), analysis metric (eg, change from baseline, final value, time to event), method of aggregation (eg, median, proportion), and time point for each outcome. Explanation of the clinical relevance of chosen efficacy and harm outcomes is strongly recommended	p.12

For peer review only - http://bmjopen.bmj.com/site/about/guidelines.xhtml

Participant timeline	13	Time schedule of enrolment, interventions (including any run-ins and washouts), assessments, and visits for participants. A schematic diagram is highly recommended (see Figure)	Table 1, p.25
Sample size	14	Estimated number of participants needed to achieve study objectives and how it was determined, including clinical and statistical assumptions supporting any sample size calculations	p.15
Recruitment	15	Strategies for achieving adequate participant enrolment to reach target sample size	p.8

Methods: Assignment of interventions (for controlled trials)

Allocation:

For peer review only - http://bmjopen.bmj.com/site/about/guidelines.xhtml

Sequence generation	16a	Method of generating the allocation sequence (eg, computer-generated random numbers), and list of any factors for stratification. To reduce predictability of a random sequence, details of any planned restriction (eg, blocking) should be provided in a separate document that is unavailable to those who enrol participants or assign interventions	p.8
Allocation concealment mechanism	16b	Mechanism of implementing the allocation sequence (eg, central telephone; sequentially numbered, opaque, sealed envelopes), describing any steps to conceal the sequence until interventions are assigned	p.8
Implementation	16c	Who will generate the allocation sequence, who will enrol participants, and who will assign participants to interventions	p.8

For peer review only - http://bmjopen.bmj.com/site/about/guidelines.xhtml

Blinding (masking)	17a	Who will be blinded after assignment to interventions (eg, trial participants, care providers, outcome assessors, data analysts), and how	p.9
	17b	If blinded, circumstances under which unblinding is permissible, and procedure for revealing a participant's allocated intervention during the trial	N/A
**Methods: Data collection, management, and analysis**			
Data collection methods	18a	Plans for assessment and collection of outcome, baseline, and other trial data, including any related processes to promote data quality (eg, duplicate measurements, training of assessors) and a description of study instruments (eg, questionnaires, laboratory tests) along with their reliability and validity, if known. Reference to where data collection forms can be found, if not in the protocol	p.12-15

	18b	Plans to promote participant retention and complete follow-up, including list of any outcome data to be collected for participants who discontinue or deviate from intervention protocols	p.15
Data management	19	Plans for data entry, coding, security, and storage, including any related processes to promote data quality (eg, double data entry; range checks for data values). Reference to where details of data management procedures can be found, if not in the protocol	p.12-15
Statistical methods	20a	Statistical methods for analysing primary and secondary outcomes. Reference to where other details of the statistical analysis plan can be found, if not in the protocol	p.16
	20b	Methods for any additional analyses (eg, subgroup and adjusted analyses)	N/A
	20c	Definition of analysis population relating to protocol non-adherence (eg, as randomised analysis), and any statistical methods to handle missing data (eg, multiple imputation)	N/A

For peer review only - http://bmjopen.bmj.com/site/about/guidelines.xhtml

Methods: Monitoring

Data monitoring	21a	Composition of data monitoring committee (DMC); summary of its role and reporting structure; statement of whether it is independent from the sponsor and competing interests; and reference to where further details about its charter can be found, if not in the protocol. Alternatively, an explanation of why a DMC is not needed	N/A – feasibility study
	21b	Description of any interim analyses and stopping guidelines, including who will have access to these interim results and make the final decision to terminate the trial	N/A
Harms	22	Plans for collecting, assessing, reporting, and managing solicited and spontaneously reported adverse events and other unintended effects of trial interventions or trial conduct	p.15 and 16
Auditing	23	Frequency and procedures for auditing trial conduct, if any, and whether the process will be independent from investigators and the sponsor	p.12

Ethics and dissemination

Research ethics approval	24	Plans for seeking research ethics committee/institutional review board (REC/IRB) approval	p.17-18
Protocol amendments	25	Plans for communicating important protocol modifications (eg, changes to eligibility criteria, outcomes, analyses) to relevant parties (eg, investigators, REC/IRBs, trial participants, trial registries, journals, regulators)	P17-18
Consent or assent	26a	Who will obtain informed consent or assent from potential trial participants or authorised surrogates, and how (see Item 32)	p.8
	26b	Additional consent provisions for collection and use of participant data and biological specimens in ancillary studies, if applicable	N/A
Confidentiality	27	How personal information about potential and enrolled participants will be collected, shared, and maintained in order to protect confidentiality before, during, and after the trial	p.8 and 18

For peer review only

Declaration of interests	28	Financial and other competing interests for principal investigators for the overall trial and each study site	p.19
Access to data	29	Statement of who will have access to the final trial dataset, and disclosure of contractual agreements that limit such access for investigators	p.19
Ancillary and post-trial care	30	Provisions, if any, for ancillary and post-trial care, and for compensation to those who suffer harm from trial participation	N/A
Dissemination policy	31a	Plans for investigators and sponsor to communicate trial results to participants, healthcare professionals, the public, and other relevant groups (eg, via publication, reporting in results databases, or other data sharing arrangements), including any publication restrictions	p.17-18
	31b	Authorship eligibility guidelines and any intended use of professional writers	p.18

For peer review only - http://bmjopen.bmj.com/site/about/guidelines.xhtml

	31c	Plans, if any, for granting public access to the full protocol, participant-level dataset, and statistical code	p.19
**Appendices**			
Informed consent materials	32	Model consent form and other related documentation given to participants and authorised surrogates	Not included in the protocol manuscript
Biological specimens	33	Plans for collection, laboratory evaluation, and storage of biological specimens for genetic or molecular analysis in the current trial and for future use in ancillary studies, if applicable	N/A

*It is strongly recommended that this checklist be read in conjunction with the SPIRIT 2013 Explanation & Elaboration for important clarification on the items. Amendments to the protocol should be tracked and dated. The SPIRIT checklist is copyrighted by the SPIRIT Group under the Creative Commons "Attribution-NonCommercial-NoDerivs 3.0 Unported" license.

For peer review only
